# Transcriptomic and DNA methylation insights into polyploidy-enhanced heat tolerance in rice (*Oryza sativa* L.)

**DOI:** 10.1093/plphys/kiag135

**Published:** 2026-03-17

**Authors:** Changjiang Zhang, Yu Wang, Weilong Meng, Xinfang Yu, Minghong Xu, Yingkai Wang, Lingxi Xiong, Xin Qi, Xintong Ma, Jian Ma, Ningning Wang

**Affiliations:** Faculty of Agronomy, Jilin Agricultural University, Changchun 130117, China; Faculty of Agronomy, Jilin Agricultural University, Changchun 130117, China; Faculty of Agronomy, Jilin Agricultural University, Changchun 130117, China; Faculty of Agronomy, Jilin Agricultural University, Changchun 130117, China; Faculty of Agronomy, Jilin Agricultural University, Changchun 130117, China; Faculty of Agronomy, Jilin Agricultural University, Changchun 130117, China; Faculty of Agronomy, Jilin Agricultural University, Changchun 130117, China; Faculty of Agronomy, Jilin Agricultural University, Changchun 130117, China; Faculty of Agronomy, Jilin Agricultural University, Changchun 130117, China; Faculty of Agronomy, Jilin Agricultural University, Changchun 130117, China; Sanjiang Laboratory, Changchun 130117, China; Faculty of Agronomy, Jilin Agricultural University, Changchun 130117, China; Jilin Provincial Laboratory of Crop Germplasm Resources, Changchun 130117, China

## Abstract

Extreme heat constrains global rice production. Polyploidy, a central driver of flowering plant evolution, is frequently associated with enhanced resilience to adverse environments. However, the epigenomic and transcriptomic programs that support heat tolerance in autotetraploid rice remain largely unexplored. In this study, we compared a diploid japonica rice line (GFD-2X) and its isogenic autotetraploid counterpart (GFD-4X) under short-term heat stress and subsequent recovery using physiological measurements, transcriptome profiling, and whole-genome DNA methylation analysis. Both cytotypes showed elevated physiological and biochemical indicators after heat treatment, with GFD 4X displaying consistently stronger responses. Transcriptome analysis revealed that heat adaptation relies mainly on hormone-related signaling pathways, heat shock proteins, and antioxidant enzyme systems. Genome-wide DNA methylation profiling revealed a contrasting pattern in which polyploidization promotes widespread DNA hypermethylation, while acute heat stress triggers broad DNA hypomethylation. This bidirectional regulatory shift suggests a dynamic feedback mechanism that contributes to environmental adaptability. Integrated analysis of methylation and gene expression further showed that heat stress reshapes the methylation patterns of stress-responsive genes, thereby altering their transcriptional regulation. Together, these results support a model in which polyploidy-associated epigenomic features and heat-induced methylation dynamics are linked to enhanced physiological and molecular responsiveness under elevated temperature. This study provides a systems-level view of how polyploid rice responds to heat stress and offers insight into the potential epigenetic basis of heat tolerance in a warming climate.

## Teaser

Polyploid rice enhances heat tolerance through dynamic epigenetic regulation, as DNA methylation patterns alter gene expression to improve physiological and molecular responses to high temperatures.

## Introduction

As a primary staple for more than half of the global population, rice (*Oryza sativa* L.) faces mounting threats from adverse climatic conditions, particularly extreme heat, which increasingly undermines yield stability and global food security. It has been estimated that rice suffers a 10% yield reduction for every one-degree Celsius increase in night temperature (Peng et al. 2004b). Polyploidization, or whole genome duplication (WGD), is one of the major pathways driving plant evolutionary innovation (Van de Peer et al. 2017; Pelé et al. 2018). Polyploid species are widespread in nature and frequently occupy broader ecological niches, particularly under stressful environmental conditions, suggesting an enhanced adaptive capacity in comparison with their diploid relatives (Van de Peer et al. 2021). Autopolyploidy, in particular, can generate phenotypic novelty directly through increased genome content and gene dosage effects, without the confounding influence of interspecific hybridization. Such novelties include increased biomass production, higher accumulation of secondary metabolites, and enhanced tolerance to environmental stress (Chen et al. 2021). For example, allopolyploid strawberry exhibits markedly greater adaptability under stress conditions than its diploid wild counterpart (Wei et al. 2019). In *Arabidopsis*, WGD-derived genes broaden expression diversity both within and between species, providing an evolutionary advantage comparable to that observed in yeast and *Drosophila*, thereby suggesting a conserved phenomenon across kingdoms (Ha et al. 2009; Van de Peer et al. 2017). Understanding how polyploid plants respond to heat stress is therefore important for elucidating stress-adaptive mechanisms and, more broadly, for safeguarding global food security in the context of a warming climate.

Heat stress adversely affects plant growth and development in a complex and multifaceted manner, with outcomes dependent on stress intensity, duration, and developmental stage (Szymańska et al. 2017). Elevated temperatures trigger stomatal closure in leaves, which restricts transpiration and ultimately leads to leaf rolling and wilting (Peng et al. 2004a). Osmoregulatory compounds help maintain cellular osmotic balance under heat-induced water deficit, while antioxidant enzymes convert reactive oxygen species (ROS) into harmless molecules, thereby protecting cells from oxidative injury (Jomova et al. 2024). Short-term osmotic stress sharply increases ROS levels, proline accumulation, and the activities of catalase (CAT) and superoxide dismutase (SOD) in maize root tips (Wang et al. 2025b). In rice, autotetraploid plants exhibit higher proline content and lower malondialdehyde (MDA) accumulation than their diploid counterparts, indicating that genome duplication strengthens root tolerance to salt stress (Wang et al. 2021).

Endogenous plant hormones play a crucial regulatory role in plant stress responses (Waadt et al. 2022). Ethylene (ETH) content is higher in tetraploid citrus than in diploid citrus. Under salt stress, polyploidization can enhance the recovery capacity of citrus plants after salt stress treatment through ETH signal transduction (Song et al. 2025b). Under drought stress, tetraploid *Arabidopsis* accumulates more abscisic acid (ABA) to cope with drought-induced damage (del Pozo and Ramirez-Parra 2014). Plants have developed various gene expression regulatory mechanisms to mitigate heat stress damage (Ohama et al. 2017). The C2H2 family transcription factor NAT1 directly inhibits the expression of *bHLH110*, which, under high-temperature stress, can directly promote the expression of the wax synthesis genes *CER1*/*CER1L*, increasing wax deposition and enhancing the heat tolerance of rice (Lu et al. 2025). *OsHTAS* expression is upregulated under high-temperature stress, and it enhances the heat tolerance of rice by regulating the expression of a series of downstream heat-response genes and participating in the scavenging of ROS. Rice plants overexpressing *OsHTAS* exhibit better growth and higher survival rates after high-temperature treatment (Liu 2015). Overexpression of the peroxidase (POD) gene *ZmPrx25* in *Arabidopsis* promotes seed germination and plant growth and significantly enhances tolerance to osmotic and oxidative stress (Zhang et al. 2025).

DNA methylation is a dynamic epigenetic mark that responds to environmental cues, modulates gene expression, and plays an essential role in plant development and stress adaptation (Zhu 2009; Law and Jacobsen 2010; Fan et al. 2022). In soybean, salt stress reduces the activity of DNA methyltransferases in seedlings, resulting in global hypomethylation and enhanced expression of ABA-dependent stress response genes, which are critical for salt tolerance (Yung et al. 2024). In *Arabidopsis*, *MYB74* suppresses seed germination under salt stress, and hypomethylation of its promoter enables salt-induced activation of *MYB74* (Xu et al. 2015). Polyploidization can also reshape DNA methylation patterns. In allotetraploid cotton, methylation marks can behave as epialleles and remain stably inherited, whereas in allohexaploid wheat, these marks may be reversed during genome separation and reunion (Zhang et al. 2015; Song et al. 2017; Yuan et al. 2020). Changes in DNA methylation have likewise been reported in autotetraploid rice, where reduced methylation levels facilitate rapid and efficient activation of jasmonic acid (JA) pathway genes under salt stress (Wang et al. 2021). Despite these advances, how DNA methylation dynamics interact with ploidy level to shape heat stress responses in rice remains poorly understood. To elucidate the differential responses of diploid and tetraploid rice under elevated temperature, we conducted an integrated analysis combining physiological measurements, transcriptome profiling, and whole-genome methylome sequencing in an isogenic diploid–autotetraploid rice pair exposed to short-term heat stress and subsequent recovery. By focusing on genome-wide patterns of transcriptional and epigenetic reprogramming rather than individual gene manipulation, this study aims to provide a system-level framework for understanding how polyploidy reshapes molecular responsiveness to heat stress in rice.

## Results

### Tetraploid rice exhibits a stronger growth advantage under heat stress

**Figure 1:**
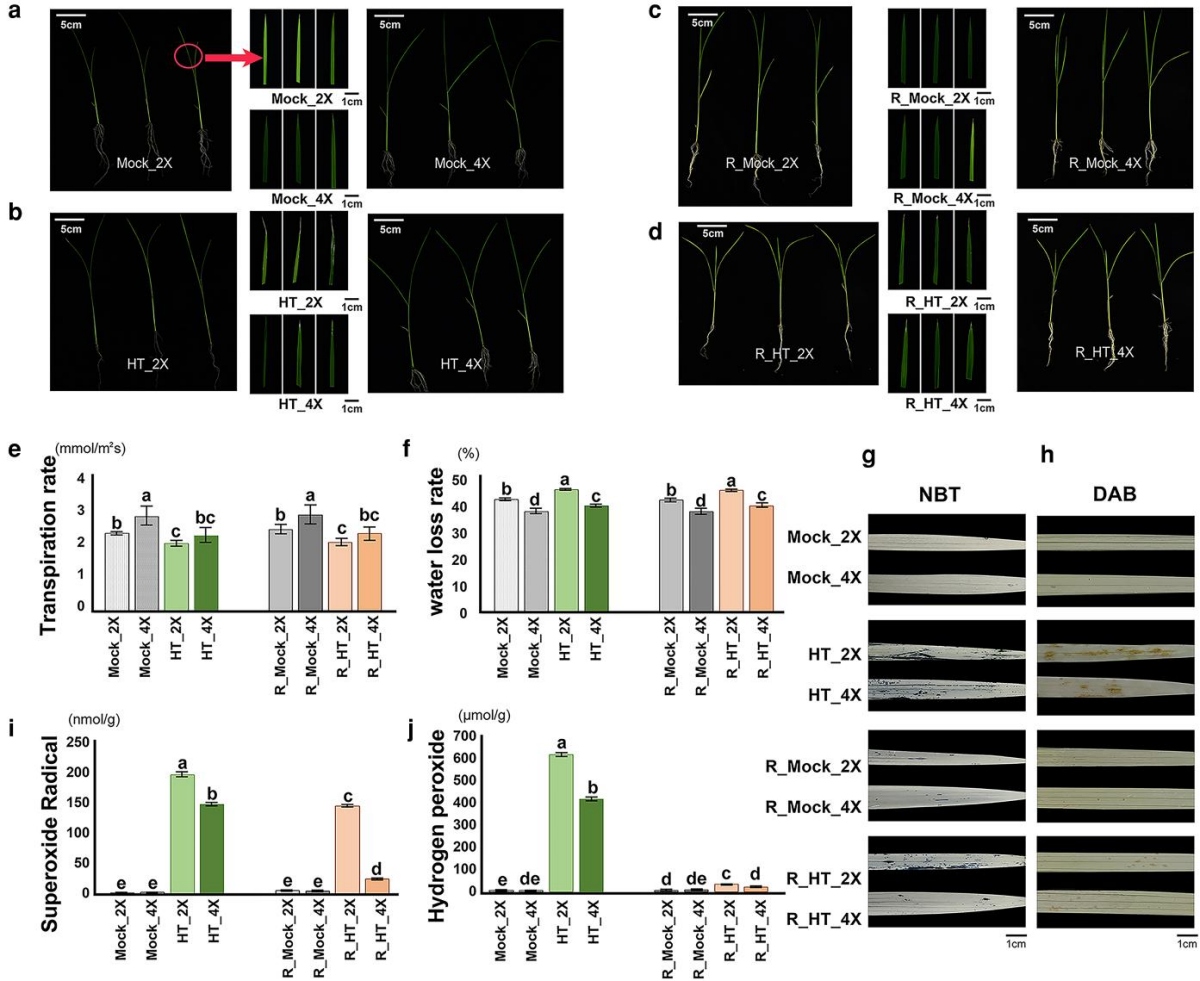
Morphological and physiological responses of diploid (GFD-2X) and tetraploid (GFD-4X) rice seedlings under heat stress. **(a)** Phenotypes of diploid (Mock_2X) and tetraploid (Mock_4X) seedlings after 7 d under normal growth conditions. Bar = 5 cm (plant), Bar = 1 cm (leaf). **(b)** Phenotypes of diploid (HT_2X) and tetraploid (HT_4X) seedlings after 7 d of heat treatment. **(c)** Phenotypes of diploid (R_Mock_2X) and tetraploid (R_Mock_4X) seedlings after 14 d under normal growth conditions. **(d)** Phenotypes of diploid (R_HT_2X) and tetraploid (R_HT_4X) seedlings after 7 d of recovery following heat stress. **(e)** Transpiration rate. **(f)** Water loss rate. **(g, h)** NBT and DAB staining of leaves from diploid and tetraploid seedlings under each treatment. Bar = 1 cm. **(i)** Superoxide radical content. **(j)** Hydrogen peroxide content. The detections were performed with 3 biological replicates, each biological replicate consisted of 3 technical replicates, with 10 plant strains per replicate. The error bar type is standard deviation. The statistical test method is one-way analysis of variance (ANOVA). Different lowercase letters (a–e) indicate statistically significant differences (*P* < 0.05). Mock_2X: Control diploid during stress. Mock_4X: Control tetraploid during stress. HT_2X: High-temperature stressed diploid. HT_4X: High-temperature stressed tetraploid. R_Mock_2X: Control diploid during recovery. R_Mock_4X: Control tetraploid during recovery. R_HT_2X: High-temperature stressed diploid during recovery. R_HT_4X: High-temperature stressed tetraploid during recovery.

Diploid rice (GFD-2X) and its isogenic autotetraploid line (GFD-4X) were subjected to heat treatment at 40 °C for 7 d, followed by a 7 d recovery period under normal growth conditions. Morphological observations showed that, compared with their respective controls, GFD-2X plants displayed pronounced leaf rolling, wilting, and chlorosis during both the heat treatment and recovery phases, whereas GFD-4X plants maintained a noticeably healthier appearance (Fig. 1a–d). Under heat stress, GFD-2X exhibited greater reductions in fresh weight, dry weight, shoot length, shoot biomass, root length, and root biomass than GFD-4X (Fig. S1). Physiologically, plants mitigate heat-induced injury by maintaining cellular water status and reducing excessive water loss to preserve protoplasmic stability (Ye et al. 1996). Under heat stress, plants initially increase transpiration to dissipate heat and maintain leaf surface temperature. However, excessive water loss can impair photosynthesis and disrupt normal metabolism. Therefore, plants must balance water uptake and water loss to sustain growth while minimizing the risk of dehydration. Consequently, heat-tolerant species often exhibit earlier stomatal closure and reduced transpiration rates under heat stress, enabling better water conservation and maintenance of normal growth (Yang et al 2020). Consistent with this principle, GFD-4X exhibited a higher transpiration rate than GFD-2X under control conditions. After heat stress treatment, the transpiration rates of both genotypes decreased, however, the reduction in GFD-4X (HT-4X vs Mock-4X) was more pronounced (20% vs 13%; Fig. 1e) compared to GFD-2X (HT-2X vs Mock-2X) . Under heat stress, GFD-4X maintained a significantly higher relative water content (Fig. S1) and a lower water loss rate (Fig. 1f) compared with GFD-2X, indicating superior water retention capacity. In addition to improved water status, heat treatment induced substantially higher accumulation of O_2_^−^ (Fig. 1i) and H_2_O_2_ (Fig. 1j) in GFD-2X plants than in GFD-4X. NBT (nitro blue tetrazolium) and DAB (diaminobenzidine) staining further confirmed that tetraploid rice possessed a stronger ROS scavenging capacity than its diploid counterpart (Fig. 1g and h). To further quantify these phenotypic differences, we performed signal intensity analysis of stained leaves. Heatmap results showed that both NBT and DAB staining intensities increased in GFD-4X and GFD-2X leaves under heat stress, however, the increase was markedly more pronounced in GFD-2X (Fig. S1). Together, morphological observations and quantitative analyses consistently demonstrate that GFD-4X exhibits enhanced tolerance to heat stress compared with GFD-2X.

### Landscape of gene expression in the response of rice to heat stress

**Figure 2:**
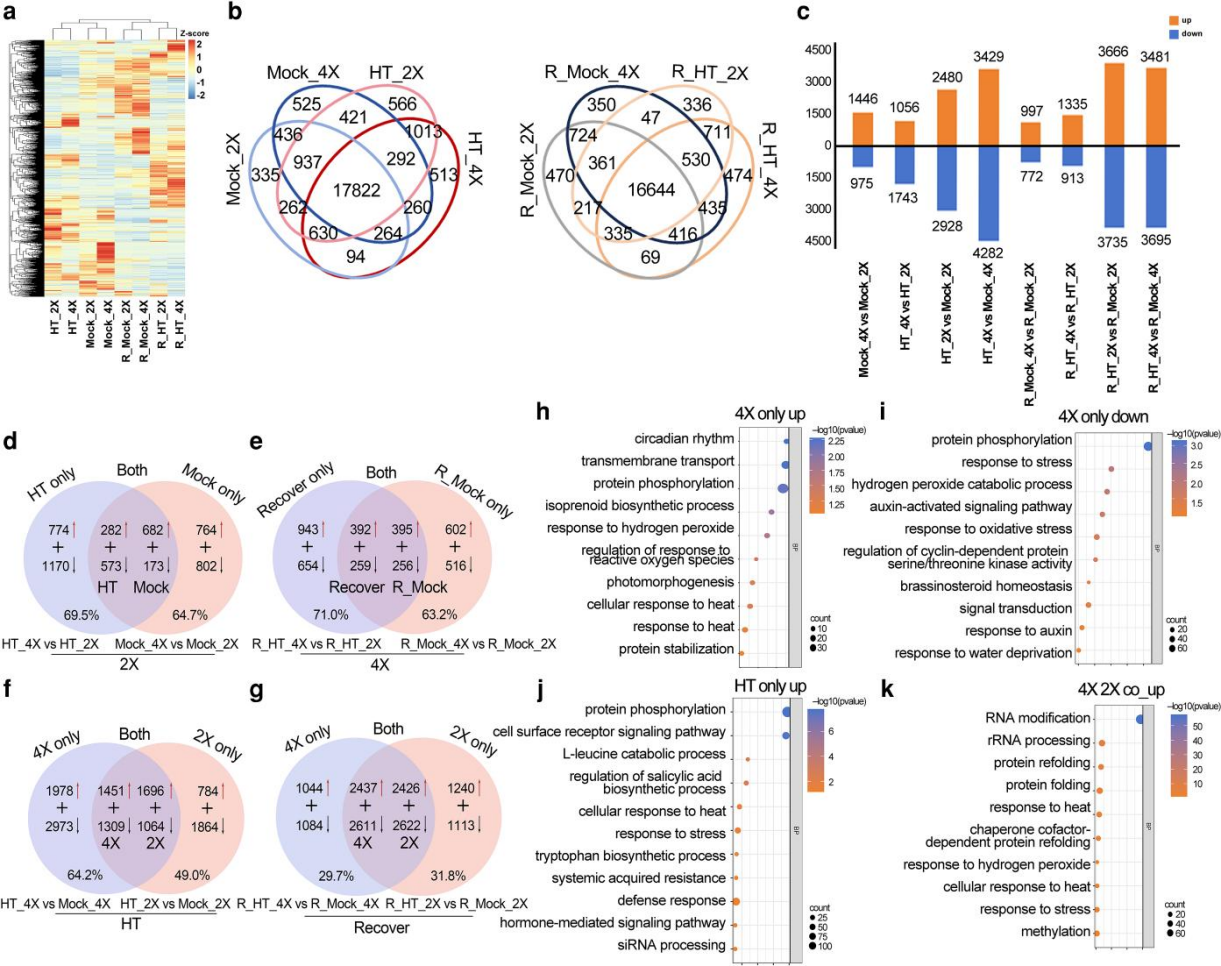
Genome-wide analysis of gene expression profiles in diploid (GFD-2X) and tetraploid (GFD-4X) rice. **(a)** Heatmap illustrating global transcriptomic characteristics of diploid and tetraploid rice under control, heat stress, and recovery conditions. **(b)** Venn diagram of expressed genes between control, heat-treated, and recovery groups, showing the number of shared and specific expressed genes under different treatments. **(c)** Bar chart displaying the number of upregulated and downregulated differentially expressed genes (DEGs) across comparison groups. The *y* axis represents the number of DEGs, with dark yellow indicating the number of upregulated genes and blue indicating the number of downregulated genes. **(d)** Venn diagram summarizing DEGs in heat stress response, showing the number of heat-stress-specific DEGs, heat-control-specific DEGs, and DEGs persistently expressed after stress treatment. **(e)** Venn diagram summarizing DEGs in recovery response, showing recovery-group-specific DEGs, recovery-control-specific DEGs, and DEGs persistently expressed after recovery treatment. **(f)** Venn diagram summarizing ploidy-dependent DEGs under heat stress, showing heat-stress-specific DEGs of tetraploid rice, heat-stress-specific DEGs of diploid rice, and heat-stress-shared DEGs. **(g)** Venn diagram summarizing ploidy-dependent DEGs under recovery, showing recovery-group-specific DEGs, recovery-control-specific DEGs, and recovery-treated-shared DEGs. **(h)** Gene Ontology (GO) enrichment analysis of heat-stress-specific upregulated DEGs in tetraploid rice. **(i)** GO enrichment analysis of heat-stress-specific downregulated DEGs in tetraploid rice. **(j)** GO enrichment analysis of DEGs specifically upregulated under heat stress. **(k)** GO enrichment analysis of co-upregulated DEGs between diploid and tetraploid rice under heat stress. 2X: diploid. 4X: tetraploid. Mock_2X: Control diploid during stress. Mock_4X: Control tetraploid during stress. HT-2X: High-temperature stressed diploid. HT_4X: High-temperature stressed tetraploid. R_Mock_2X: Control diploid during recovery. R_Mock_4X: Control tetraploid during recovery. R_HT_2X: High-temperature stressed diploid during recovery. R_HT_4X: High-temperature stressed tetraploid during recovery.

To elucidate the molecular basis underlying the enhanced heat tolerance of GFD-4X, transcriptome profiling was conducted for GFD-2X and GFD-4X leaves under control, heat stress, and recovery conditions, with 3 biological replicates for each treatment (Table S1). Cluster analysis showed that GFD-2X and GFD-4X samples grouped together under both normal and heat stress conditions, indicating that gene expression patterns remained similar between ploidy levels when exposed to the same environment (Fig. 2a). Venn diagram analysis revealed that GFD-2X and GFD-4X possessed 335 and 525 uniquely expressed genes under control and heat stress conditions, and 566 and 513 uniquely expressed genes under heat stress, respectively. Under recovery conditions, 470 and 350 uniquely expressed genes were detected in GFD-2X and GFD-4X control samples, and 336 and 474 in the stress recovery samples (Fig. 2b). Differentially expressed genes (DEGs) were identified using the criteria count > 30, |log_2_ (fold change) | > 1, and *P*-value < 0.05 (Table S2). GFD-4X displayed a greater number of upregulated and downregulated genes than GFD-2X under heat stress. In total, 5,408 and 7,711 DEGs were detected in GFD-2X and GFD-4X under heat treatment, respectively, and these DEGs were defined as heat stress–responsive genes (Fig. 2c). Among these, 2,480 genes were upregulated and 2,928 downregulated in diploid rice, whereas tetraploid rice exhibited 3,429 upregulated and 4,282 downregulated genes (Fig. 2c).

A substantial proportion of DEGs between tetraploid and diploid rice showed conserved expression patterns under both control and heat stress conditions (Fig. 2d). Specifically, 55.8% [(282 + 682)/(282 + 682 + 764)] of upregulated genes and 48.2% [(573 + 173)/(573 + 173 + 802)] of downregulated genes under control conditions maintained their expression direction after heat treatment, indicating that a core regulatory framework of stress-induced gene expression is conserved across ploidy levels (Fig. 2d). A distinct group of genes was differentially expressed between GFD-2X and GFD-4X specifically under heat stress, with 44.5% [774/(774 + 282 + 682)] classified as upregulated and 61.1% [1,170/(1,170 + 573 + 173)] classified as downregulated (Fig. 2d). These genes were designated as ploidy-dependent stress-responsive genes.

A similar pattern was observed when stress-responsive expression was evaluated independently within GFD-4X. Under heat stress, the expression of a large number of DEGs remained stable in both GFD-2X and GFD-4X (Fig. 2f). In GFD-4X, 80.1% [(1,451 + 1,696)/(1,451 + 1,696 + 784)] of upregulated genes and 56.1% [(1,309 + 1,064)/(1,309 + 1,064 + 1,864)] of downregulated genes preserved their original expression trends after heat treatment, suggesting that the mechanisms governing stress-responsive transcription are shared between the two ploidy levels (Fig. 2f). In GFD-4X specifically, 38.6% [1,978/(1,978 + 1,451 + 1,696)] of genes were uniquely upregulated and 55.6% [2,973/(2,973 + 1,309 + 1,064)] were uniquely downregulated under heat stress (Fig. 2f). These genes were defined as polyploid-dependent stress-responsive genes.

A large proportion of DEGs displayed stable expression patterns between GFD-2X and GFD-4X under both recovery and control conditions (Fig. 2e). In the control group, 56.7% [(392 + 395)/(392 + 395 + 602)] of upregulated genes and 50.0% [(259 + 256)/(259 + 256 + 516)] of downregulated genes retained their original expression patterns after recovery, indicating that the regulatory systems controlling gene expression exhibit substantial stability following heat stress in both ploidy types (Fig. 2e). Under recovery conditions, distinct ploidy-specific differences were detected, with 54.5% [943/(943 + 392 + 395)] of genes classified as upregulated and 55.9% [654/(654 + 259 + 256)] classified as downregulated (Fig. 2e). These genes were defined as heat stress recovery-responsive genes. A similar trend was evident when expression stability was examined within each ploidy level (Fig. 2g). In tetraploid rice, 79.3% [(2,437 + 2,426)/(2,437 + 2,426 + 1,240)] of upregulated genes and 82.4% [(2,611 + 2,622)/(2,611 + 2,622 + 1,113)] of downregulated genes preserved their expression direction following recovery (Fig. 2g). A distinct subset of genes showed ploidy-specific expression during recovery, with 17.6% [1,044/(1,044 + 2,437 + 2,426)] classified as uniquely upregulated and 17.1% [1,084/(1,084 + 2,611 + 2,622)] classified as uniquely downregulated in GFD-4X (Fig. 2g). These genes were designated as polyploid-dependent heat stress recovery genes. Collectively, these data indicate that polyploidization has strengthened the robustness of gene expression responses during and after heat stress.

To further elucidate the molecular mechanisms underlying the short-term heat stress response in rice with different ploidy levels, gene ontology (GO) enrichment analysis was performed on the identified DEGs (Fig. S2; Table S3). In the comparisons HT_4X vs. Mock_4X and HT_2X vs. Mock_2X, the 1,978 genes specifically upregulated by heat stress only in GFD-4X were significantly enriched in GO terms associated with responses to hydrogen peroxide (H_2_O_2_), regulation of ROS, cellular responses to heat, heat stress responses, and protein stabilization (Fig. 2h). Conversely, the 2,973 tetraploid-specific downregulated genes were significantly enriched in GO terms including protein phosphorylation, response to stress, H_2_O_2_ catabolic process, auxin-activated signaling pathways, and response to oxidative stress (Fig. 2i). In the comparisons HT_4X vs. HT_2X and Mock_4X vs. Mock_2X, the 774 genes specifically induced and upregulated only in GFD-4X under heat stress showed significant enrichment in GO categories related to protein phosphorylation, cell surface receptor signaling pathways, L-leucine catabolic processes, and regulation of salicylic acid biosynthetic processes (Fig. 2j). Meanwhile, the DEGs that were upregulated in both GFD-2X and GFD-4X plants under heat stress were significantly enriched in GO terms associated with RNA modification, response to heat, response to H_2_O_2_, and methylation (Fig. 2k). These results indicate that both GFD-2X and GFD-4X undergo complex physiological and biochemical adjustments during heat stress, potentially involving modulation of hormone signaling, antioxidant enzyme activity, and DNA methylation dynamics. Further investigation will be required to clarify these regulatory processes.

### Identification and expression pattern analysis of the candidate genes

**Figure 3:**
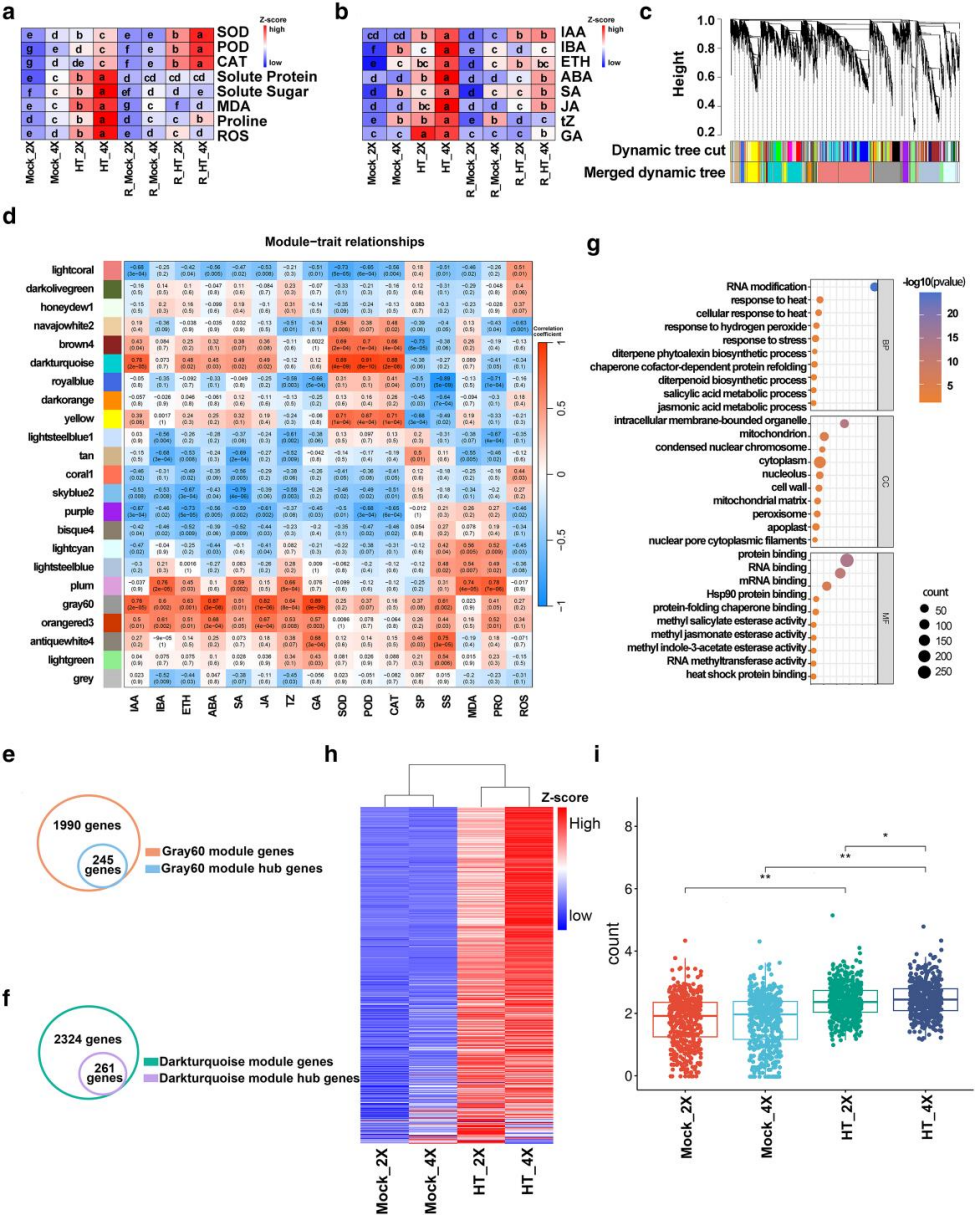
Identification of hub genes through weighted gene co-expression network analysis (WGCNA). **(a, b)** Heatmaps showing the contents of physiological indicators **(a)** and phytohormones **(b)**. Different lowercase letters (a–g) indicate statistically significant differences (*P* < 0.05). **(c)** Hierarchical clustering dendrogram of genes based on the co-expression network. Each leaf represents one gene. Genes not assigned to any co-expression module were grouped into the gray module. **(d)** Module–trait correlation heatmap. Values represent Pearson correlation coefficients between module eigengenes and physiological or hormonal traits, corresponding *P*-values are shown in parentheses. **(e, f)** Identification of hub genes in the gray60 module **(e)** and dark turquoise module **(f)** The total number of genes in each module and the number of hub genes are indicated. **(g)** Gene Ontology (GO) enrichment analysis of the identified hub genes. **(h)** Expression patterns of hub genes in diploid and tetraploid rice under different treatments. **(i)** Distribution of hub-gene expression across samples. The horizontal bar indicates the median, box edges represent the first and third quartiles. Twenty plants were pooled together as one sample in each replicate. All samples were tested in 3 independent experiments with 3 replicates each. The statistical test method is one-way analysis of variance (ANOVA). Single and double asterisks denote significance at *P* < 0.05 and *P* < 0.01, respectively. Superoxide Dismutase: SOD, Peroxidase: POD, Catalase: CAT, Solute Protein: SP, Solute Sugar: SS, Malondialdehyde: MDA, Proline: PRO, Reactive Oxygen Species: ROS, Indole-3-acetic acid: IAA, Indole-3-butyric acid: IBA, Ethylene: ETH, Abscisic Acid: ABA, Salicylic Acid: SA, Jasmonic Acid: JA, Zeatin: tZ, Gibberellin: GA. Mock_2X: Control diploid during stress. Mock_4X: Control tetraploid during stress. HT_2X: High-temperature stressed diploid. HT_4X: High-temperature stressed tetraploid. R_Mock_2X: Control diploid during recovery. R_Mock_4X: Control tetraploid during recovery. R_HT_2X: High-temperature stressed diploid during recovery. R_HT_4X: High-temperature stressed tetraploid during recovery.

To investigate more deeply how heat stress affects the physiological and biochemical traits of GFD-2X and GFD-4X, a series of antioxidant-related physiological indicators, including SOD, POD, CAT, soluble proteins, soluble sugars, MDA, proline, and ROS, were measured (Fig. 3a). Under heat stress, soluble proteins, soluble sugars, MDA, proline, and ROS levels were all markedly elevated. These increases were more pronounced in GFD-4X than in GFD-2X. During the recovery phase, the levels of these indicators declined and returned to values similar to their respective controls. In contrast, the activities of SOD, POD, and CAT increased significantly during recovery, with GFD-4X showing the strongest induction. Under heat stress, GFD-4X also displayed significant increases in the activities of these antioxidant enzymes, whereas the GFD-2X plans exhibited little change. Phytohormone contents, including indole-3-acetic acid (IAA), indole-3-butyric acid (IBA), ETH, ABA, salicylic acid (SA), JA, trans-zeatin (tZ), and gibberellin (GA), were also quantified (Fig. 3b). All hormones exhibited strong fluctuations during heat treatment. Both GFD-2X and GFD-4X showed significant increases in hormone levels under heat stress, with GFD-4X exhibiting consistently higher concentrations than GFD-2X. During recovery, hormone levels decreased, however, in GFD-4X, IAA, ABA, SA, and JA levels remained significantly higher than their respective controls.

### Weighted gene co-expression network analysis of heat-responsive genes

Weighted gene co-expression network analysis (WGCNA) was performed using the fragments per kilobase of transcript per million mapped reads (FPKM) values of 15,335 DEGs identified in GFD-2X and GFD-4X under control and heat-stress conditions, together with 16 phenotypic traits. A total of 15,320 DEGs were assigned to 23 co-expression modules (Fig. 3c). Among these, the “gray60” and “dark turquoise” modules showed the strongest correlations with the hormone traits IAA, ABA, ETH, JA, and GA (Fig. 3d). Within the “gray60” and “dark turquoise” modules, 1,990 and 2,324 DEGs were identified, yielding 245 and 261 hub genes, respectively (Figs. 3e and 3f). GO enrichment analysis of these hub genes showed significant enrichment in pathways including response to heat, response to H_2_O_2_, chaperone cofactor-dependent protein refolding, SA metabolic processes, and JA metabolic processes (Fig. 3g).

Heatmap analysis demonstrated that the expression patterns of these hub genes across samples were consistent with the trends observed in the physiological and biochemical indicators (Fig. 3h; Table S4). Boxplot analysis further showed that under control conditions, hub gene expression did not differ significantly between GFD-2X and GFD-4X. After heat treatment, however, expression levels were markedly upregulated in both cytotypes, with GFD-4X exhibiting substantially higher expression than GFD-2X (Fig. 3i).

### Polyploidization induces DNA hypermethylation, whereas heat stress promotes hypomethylation

Previous studies have shown that DNA methylation plays an important regulatory role in plant responses to heat (Yin et al. 2024). To examine methylation dynamics in rice, methylome sequencing was performed on leaves of GFD-2X and GFD-4X plants under control and heat-stress conditions. The sequencing results showed that all samples had Q20 values above 97% and Q30 values above 93%, with an average coverage of approximately tenfold (cytosine coverage around 83%). In total, 96,786,988 cytosines were identified, representing about 70% of all cytosines in the rice genome (Table S1).

**Figure 4:**
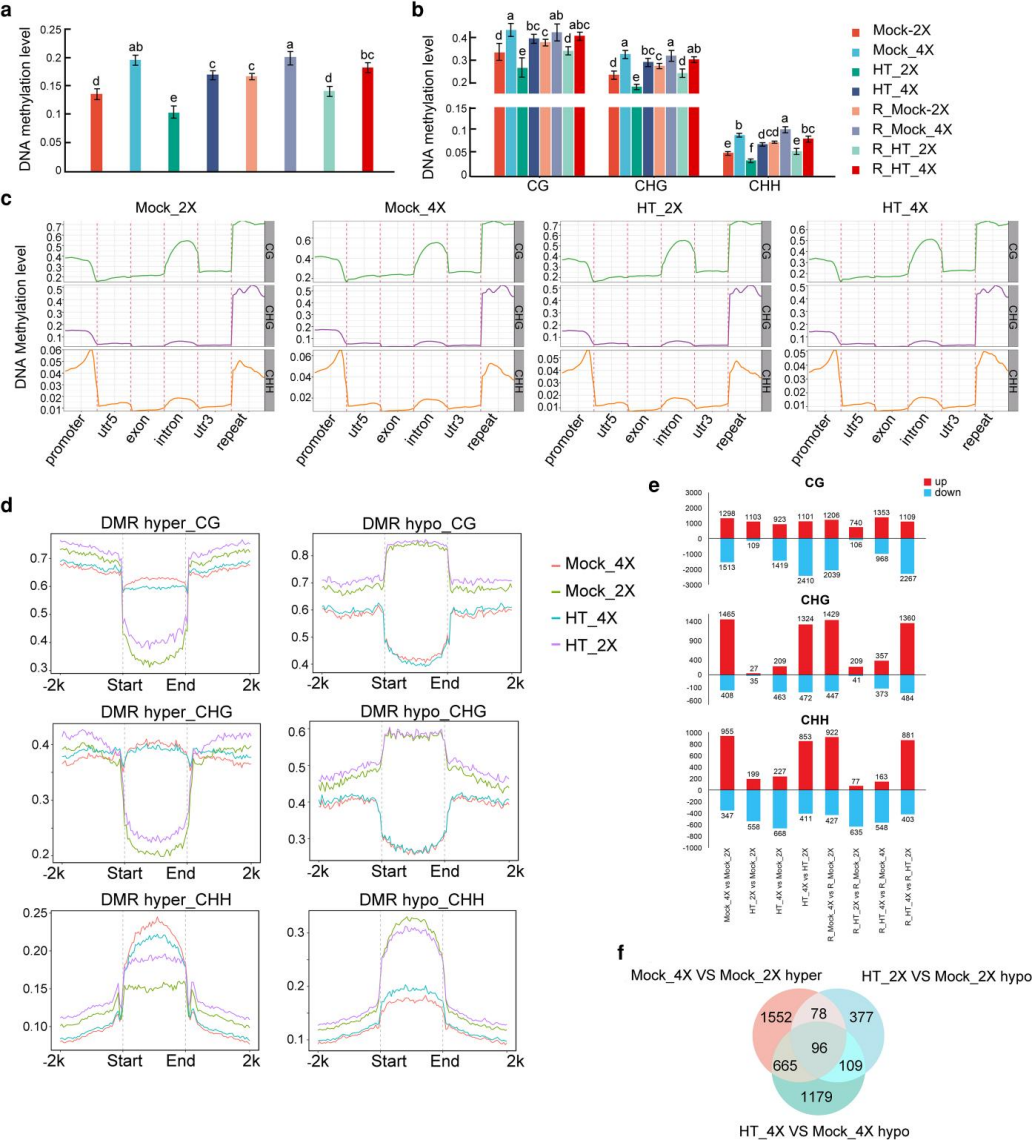
DNA methylation profiling of diploid (GFD-2X) and tetraploid (GFD-4X) rice under heat stress. **(a, b)** Global DNA methylation levels **(a)** and methylation levels of CG, CHG, and CHH contexts **(b)**. Twenty plants were pooled together as one sample in each replicate. All samples were tested in 3 independent experiments with 3 replicates each. Error bars represent standard deviation, statistical analysis was performed using one-way analysis of variance (ANOVA). Different lowercase letters (a–f) indicate statistically significant differences (*P* < 0.05). **(c)** Distribution of DNA methylation levels across genomic features, including promoters, 5′untranslated regions (UTRs), exons, introns, 3′UTRs, and repetitive regions, in diploid and tetraploid rice under different treatments. **(d)** Methylation profiles of hypermethylated and hypomethylated differentially methylated regions (DMRs) in CG, CHG, and CHH contexts across diploid and tetraploid samples. **(e)** Numbers of hyper- and hypomethylated DMRs for each methylation context (CG, CHG, CHH) across the comparisons between diploid and tetraploid rice under control, heat, and recovery conditions. The *y* axis represents the number of DMRs. **(f)** Venn diagram showing the overlap between ploidy-associated hyper-DMRs and heat-induced hypo-DMRs. Mock_2X: Control diploid during stress. Mock_4X: Control tetraploid during stress. HT_2X: High-temperature stressed diploid. HT_4X: High-temperature stressed tetraploid. R_Mock_2X: Control diploid during recovery. R_Mock_4X: Control tetraploid during recovery. R_HT_2X: High-temperature stressed diploid during recovery. R_HT_4X: High-temperature stressed tetraploid during recovery.

Overall, global DNA methylation levels in the CG, CHG, and CHH contexts decreased markedly in both GFD-2X and GFD-4X plants under heat stress compared with their respective controls (Figs. 4a and 4b). Under heat treatment, methylation levels in GFD-4X decreased by 8.6%, 10.9%, and 22% in CG, CHG, and CHH contexts, respectively, while GFD-2X showed reductions of 20.7%, 22.9%, and 32.8%. After recovery, DNA methylation in GFD-2X remained significantly lower than in the control, whereas GFD-4X did not exhibit a significant difference compared with their control. Under identical growth conditions, GFD-4X consistently showed higher methylation levels than GFD-2X (Fig. 4b). These findings indicate that heat stress induces genome-wide hypomethylation in rice.

To characterize methylation variation in functional genomic regions, DNA methylation profiles were examined across all cytosine contexts in promoters, gene bodies, and transposable element (TE) regions, revealing distinct patterns between ploidy levels under both control and stress conditions (Fig. 4c; Fig. S3). Compared with the control, promoter methylation levels decreased in both GFD-2X and GFD-4X after stress, with a greater reduction observed in GFD-4X. This promoter hypomethylation is consistent with the enhanced gene expression detected after heat stress, particularly in GFD-4X. Under normal conditions, higher CG methylation was observed in GFD-4X than in GFD-2X within gene bodies, particularly in exonic regions. After heat stress, however, methylation levels in GFD-4X were reduced below those of GFD-2X in both gene body and TE regions (Fig. 4d; Fig. S3). Given that gene body hypermethylation is generally associated with repression of gene expression, this reduction is consistent with the observed decrease in upregulated genes and increase in downregulated genes in GFD-4X relative to GFD-2X after stress.

To clarify how DNA methylation contributes to gene expression changes, differentially methylated regions (DMRs) associated with both ploidy (tetraploid vs. diploid) and stress conditions were identified (Fig. 4e; Table S5). In accordance with the global methylation patterns shown in Fig. 4b, substantially more hypo-DMRs were detected in GFD-4X (1,419, 463, and 668 in the CG, CHG, and CHH contexts, respectively) than in GFD-2X (109, 35, and 558 in the CG, CHG, and CHH contexts, respectively) under heat stress (Fig. 4e). Among the 2,391 hyper-DMRs (1,552 + 665 + 78 + 96) identified under control conditions, 761 (665 + 96) transitioned to hypo-DMRs in GFD-4X following heat stress. In contrast, only 174 hyper-DMRs (7.2% of the same total) shifted to hypo-DMRs in GFD-2X under heat stress. These hyper-DMRs in tetraploid rice under control conditions and their conversion to hypo-DMRs after heat treatment were defined as stress-related DMRs (Fig. 4f).

### Integrative analysis of transcriptome and methylome involved in key regulatory pathways

Analysis of DEGs indicated that GFD-4X exhibited more pronounced transcriptional changes than GFD-2X under heat treatment. The methylome analysis further revealed extensive reprogramming of CG and CHH methylation in GFD-4X. A comparison between stress-related differentially methylated genes (DMGs) and the hub genes identified through WGCNA led to the identification of 26 stress-related hub DMGs (Table S6). These genes were closely associated with the biosynthesis and metabolism of hormones, heat shock proteins (HSPs), and ROS, suggesting that they may play critical roles in the adaptation to elevated temperatures.

**Figure 5:**
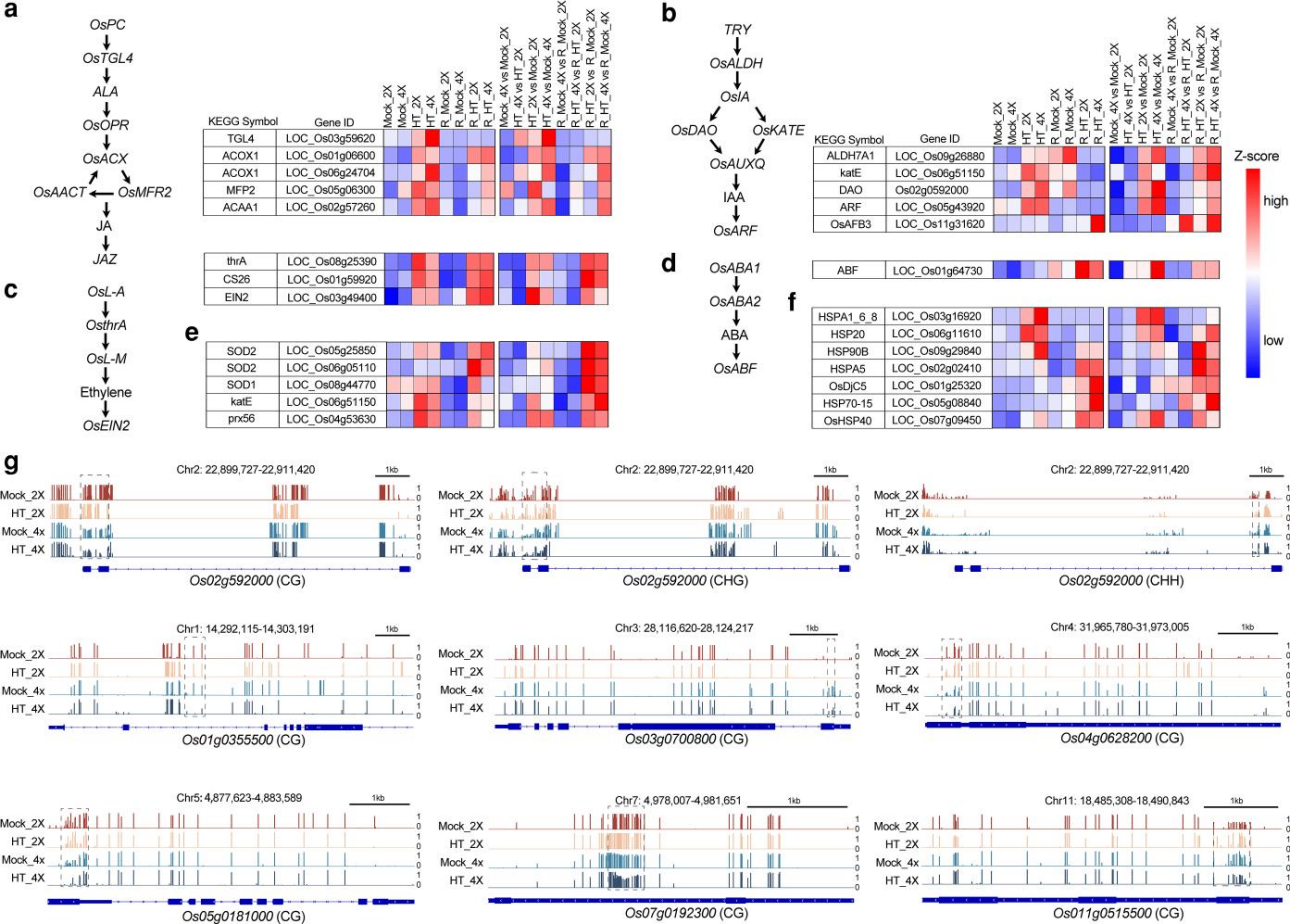
Hypomethylation-mediated regulation of stress-responsive gene expression under heat stress. **(a)** Expression patterns of genes involved in the jasmonic acid (JA) biosynthesis and signaling pathway under heat stress. Pathway schematics, heatmaps of gene expression, and associations with JA content are shown. **(b)** Expression patterns of auxin (IAA) biosynthesis and signaling genes, including key enzymes and transcriptional regulators. **(c)** Expression of ethylene biosynthesis and signaling genes under heat treatment. **(d)** Expression of ABA-related biosynthesis and signaling genes under heat stress. Panels (a–d) include pathway schematics, gene expression profiles, and the relationship between hormone levels and gene transcription. **(e)** Transcriptional analysis of reactive oxygen species (ROS)-associated genes in diploid and tetraploid rice after heat stress. **(f)** Expression profiles of heat shock protein (HSP)-related genes in response to heat stress. **(g)** Genome browser tracks showing methylation changes at representative stress-responsive genes across CG, CHG, and CHH contexts. Dashed black boxes mark differentially methylated regions (DMRs). Mock_2X: Control diploid during stress. Mock_4X: Control tetraploid during stress. HT_2X: High-temperature stressed diploid. HT_4X: High-temperature stressed tetraploid. R_Mock_2X: Control diploid during recovery. R_Mock_4X: Control tetraploid during recovery. R_HT_2X: High-temperature stressed diploid during recovery. R_HT_4X: High-temperature stressed tetraploid during recovery.

Because hormone levels increased markedly after heat treatment, the expression patterns of key genes involved in hormone biosynthesis and signaling pathways were examined to elucidate the underlying regulatory mechanisms. In the alpha-linolenic acid metabolism pathway, the genes *LOC_Os03g59620, LOC_Os01g06600, LOC_Os06g24704, LOC_Os05g06300*, and *LOC_Os02g57260* showed increased expression following heat treatment (Fig. 5a). Alpha-linolenic acid serves as the precursor of JA, suggesting that heat stress may promote JA synthesis by regulating this pathway.

In auxin signaling, the tryptophan metabolism-related genes *LOC_Os09g26880, LOC_Os06g51150, Os02g0592000*, and *LOC_Os11g31620* were upregulated after heat treatment. In addition, *LOC_Os05g43920*, a member of the ARF gene family, also showed increased expression (Fig. 5b). In the cysteine and methionine metabolism pathway, *LOC_Os08g25390* and *LOC_Os01g59920* displayed elevated expression following heat treatment. Moreover, *LOC_Os03g49400*, a key regulatory gene in the ETH signaling pathway, was upregulated, implying that ETH signaling may contribute to heat adaptation (Fig. 5c). In the plant hormone signal transduction pathway, *LOC_Os01g64730*, a member of the ABF transcription factor gene family, was also upregulated after heat treatment. Given the established role of ABF factors in abiotic stress responses, this expression pattern indicates their likely involvement in heat adaptation (Fig. 5d).

Peroxisomes constitute a central metabolic pathway for cellular detoxification and play a crucial role in the scavenging of ROS. Under heat stress, SOD catalyzes the conversion of superoxide anions into hydrogen peroxide, which is subsequently detoxified in peroxisomes enriched with CAT and POD. These enzymes further decompose hydrogen peroxide into water, thereby forming an integrated and efficient ROS-scavenging system. In this study, several genes involved in this peroxisome-associated detoxification pathway, including *LOC_Os05g25850*, *LOC_Os06g05110*, *LOC_Os07g46990*, *LOC_Os04g59200*, *LOC_Os08g44770*, *LOC_Os06g51150*, and *LOC_Os04g53630*, were significantly upregulated following heat stress treatment (Fig. 5e; Fig. S4).

Further analysis of HSP-related genes revealed that *LOC_Os03g16920, LOC_Os06g11610, LOC_Os09g29840, LOC_Os02g02410, LOC_Os01g25320, LOC_Os05g08840*, and *LOC_Os07g09450* play important roles in protein processing within the endoplasmic reticulum (ER). Proper ER function is essential for maintaining cellular homeostasis. Under heat treatment, these genes were upregulated (Fig. 5f), suggesting that they may enhance the capacity of the ER for protein folding, modification, and trafficking, thereby alleviating ER stress triggered by elevated temperatures. When the relationship between DNA methylation and gene expression was examined, methylation levels were found to decrease after heat treatment in both diploid and tetraploid rice, with a more pronounced reduction observed in tetraploid plants (Fig. 5g). This pattern suggests that DNA methylation participates in regulating the transcriptional activity of these key genes. Notably, the differentially methylated sites were not restricted to a single genomic region, indicating that methylation-mediated regulation of gene expression is a complex process. Validation of the RNA sequencing (RNA-seq) results using reverse transcription quantitative PCR (RT-qPCR) was performed for 12 selected genes (*Os01g0102900, Os01g0182600, Os05g0343400*, and others). The RT-qPCR results confirmed that the expression trends of all 8 genes were consistent with the RNA-seq data (Fig. S4).

## Discussion

**Figure 6:**
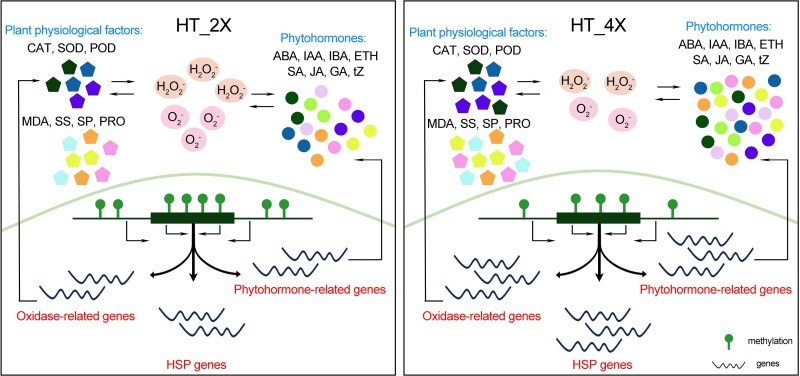
Proposed regulatory model of heat-stress responses in diploid (GFD-2X) and tetraploid (GFD-4X) rice. Heat stress induces genome-wide DNA hypomethylation, with tetraploid rice exhibiting more pronounced demethylation in key regulatory regions. This epigenetic shift enhances the transcriptional activation of stress-responsive genes, including those involved in hormone biosynthesis and signaling, heat shock protein (HSP) production, and reactive oxygen species (ROS)-scavenging pathways. Elevated expression of these genes contributes to increased levels of phytohormones, antioxidant enzymes, osmolytes, and HSPs, collectively promoting improved thermotolerance in tetraploid rice compared with diploid rice. The pentagons represent Malondialdehyde (MDA), Solute Sugar (SS), Solute Protein (SP), Proline (PRO), Catalase (CAT), Superoxide Dismutase (SOD), and Peroxidase (POD), respectively. The circles represent Abscisic Acid (ABA), Indole-3-butyric acid (IBA), Indole-3-acetic acid (IAA), Ethylene (ETH), Salicylic Acid (SA), Jasmonic Acid (JA), Gibberellin (GA), and Zeatin (tZ), respectively. HT_2X: High-temperature stressed diploid. HT_4X: High-temperature stressed tetraploid.

Understanding how polyploid plants respond to acute heat stress is essential for elucidating the mechanisms that contribute to environmental resilience. In this study, clear physiological, morphological, and molecular distinctions were observed between diploid and tetraploid rice exposed to high temperatures. The evidence demonstrated that GFD-4X maintains superior growth and physiological stability compared with GFD-2X under heat stress, highlighting the adaptive advantages conferred by genome doubling. Experimental results further showed that heat treatment was associated with hypomethylation of key stress-responsive genes in GFD-4X. This epigenetic shift was associated with enhanced accumulation of hormones, antioxidant metabolites, and HSPs—molecular components central to cellular protection and recovery (Fig. 6). These findings provide mechanistic insight into how tetraploid rice integrates transcriptional, epigenetic, and physiological responses to mitigate heat-induced damage.

### Tetraploid rice exhibits enhanced physiological resilience under heat stress

Polyploid plants have repeatedly been shown to exhibit stronger tolerance to challenging environments. For instance, polyploid Dianthus maintains superior growth performance under short-term extreme temperatures (López-Jurado et al. 2024). Consistent with this pattern, the present study revealed pronounced morphological differentiation between GFD-2X and GFD-4X under heat stress. GFD-4X displayed greater stability in dry weight, fresh weight, and plant height, coupled with higher relative water content and reduced water-loss rates. Notably, under heat stress, GFD-4X exhibited a more pronounced reduction in transpiration rate, thereby effectively limiting water loss. Collectively, these traits suggest that GFD-4X sustains water homeostasis more efficiently during heat exposure, thereby limiting cellular damage and supporting enhanced thermotolerance.

### Polyploidy strengthens the transcriptional response to heat stress

Transcriptome analyses have provided valuable insight into the molecular regulation of heat tolerance in rice. Ma et al. reported that the heat-responsive UDP-glucosyltransferase gene *OsUGT72F1* enhances thermotolerance by improving ROS scavenging capacity and modulating multiple metabolic pathways under the regulation of the transcription factor genes *OsHSFA3* and *OsHSFA4a* (Ma et al. 2025). Similarly, Li et al. demonstrated that heat stress induces the expression of genes encoding HSP factors, which subsequently mediate downstream protein folding processes (Li et al. 2024b).

In the present study, transcriptome profiling revealed that GFD-2X and GFD-4X displayed broadly similar gene expression patterns under the same treatment conditions. However, the number of genotype-specific genes differed markedly between ploidy levels. Under heat stress, the number of unique genes increased in GFD-2X but remained relatively stable in GFD-4X, after recovery, the number decreased in GFD-2X but increased in GFD-4X. These patterns suggest that GFD-2X and GFD-4X may deploy distinct molecular strategies in their response to heat stress.

Differences were also observed in the number of DEGs across comparison groups. Comparisons between control and heat-treated samples yielded a plethora of DEGs with broad expression changes. In contrast, the number of DEGs detected between GFD-2X and GFD-4X was smaller and more stable across conditions. This pattern indicates that environmental perturbation exerts a stronger influence on gene expression than ploidy itself and that genomic homology likely contributes to the largely conserved heat-responsive transcriptional framework shared by the 2 cytotypes.

Following heat stress, many upregulated genes maintained elevated expression in both GFD-2X and GFD-4X, and a subset remained upregulated even after recovery. This observation parallels our earlier findings that salt-induced stress-responsive genes in GFD-4X remain highly expressed after a return to normal growth conditions (Wang et al. 2021), suggesting short-term persistence of stress-responsive transcription following recovery, rather than stable epigenetic inheritance.

Although the GO terms enriched for GFD-4X-specific upregulated DEGs overlapped extensively with those enriched for DEGs shared between both ploidy levels, GFD-4X activated a larger number of genes within these pathways. This indicates that, while the fundamental heat-responsive mechanisms are highly similar between GFD-2X and GFD-4X, the tetraploid genotype possesses a more robust or scalable transcriptional response capacity, consistent with its enhanced physiological performance under heat stress. Physiological and biochemical components form the foundation of plant life processes, collectively constituting a highly coordinated regulatory network that determines growth, development, stress tolerance, and ultimately productivity (Luo et al. 2023). In previous studies, heat stress was shown to substantially increase H_2_O_2_ accumulation in rice (Guan 2023). It was further demonstrated that proline levels rise markedly after heat exposure, thereby enhancing thermotolerance and reducing heat-induced cellular injury (Ali and Gelani 2016). The results of the present study are consistent with these findings. Following heat treatment, GFD-4X displayed significantly higher levels of key physiological and biochemical indicators than GFD-2X. During the recovery phase, most indicators returned to control levels, however, SOD, POD, and CAT activities increased significantly in both cytotypes, with a stronger response observed in GFD-4X.

### Hormonal and antioxidant pathways underpin enhanced thermotolerance in tetraploid rice

Hormone quantification revealed that all measured phytohormones increased sharply during heat exposure and declined during recovery. Notably, in GFD-4X, the levels of IAA, ABA, SA, and JA remained significantly higher than in the control even after recovery. Together, these observations suggest that GFD-4X initiates a more vigorous physiological and hormonal response under heat stress compared with GFD-2X, enabling more effective mitigation of heat-induced damage. During recovery, GFD-4X appears to remove harmful metabolites more rapidly while maintaining elevated levels of specific stress-related hormones, potentially facilitating a faster return to normal growth and metabolic homeostasis. In peony, antioxidant enzyme activities and osmotic adjustment compounds increase significantly under high-temperature conditions, with SOD identified as the major determinant shaping physiological and biochemical variation across stress durations (Wang et al. 2022). In maize, heat stress reduces kernel weight and yield, whereas exogenous ABA application mitigates heat-induced damage during grain development (Liu et al. 2023).

In the present study, tetraploid-specific upregulated DEGs under heat stress were significantly enriched in GO terms associated with SA biosynthesis, heat response, and defense response, indicating that rice likely employs hormone-mediated signaling and HSPs to alleviate heat-induced injury. Consistent increases in physiological and hormonal indicators were observed in both cytotypes following heat treatment, with GFD-4X exhibiting more pronounced changes. These findings suggest that the synthesis and turnover of physiological and biochemical metabolites are highly responsive to environmental fluctuations, and that both polyploidization and heat stress jointly influence the expression of related genes. The WGCNA-based co-expression network further identified core genes whose expression patterns closely paralleled changes in physiological and biochemical traits. These genes were strongly upregulated after heat treatment in both GFD-2X and GFD-4X, suggesting a close association with the production of stress-related metabolites and demonstrating that their transcription is strongly heat-inducible. These observations align with previous findings. In jujube, it was reported that high temperatures significantly altered ABA and ETH contents and increased the activity of key enzymes involved in their metabolism, with elevated temperatures promoting the accumulation of ABA, ETH, and related metabolites (Wang et al. 2025a).

### Genome-wide methylation reprogramming contributes to heat-adaptive gene regulation

DNA methylation is a key epigenetic mechanism underlying gene regulation in plants in response to environmental stress. Environmental stimuli such as temperature and light frequently induce epigenetic reprogramming, and shifts in these external factors can reshape the distribution of DNA methylation across functional genomic regions, thereby modulating the transcriptional activity of regulatory genes (Lämke and Bäurle 2017). In a study by Li et al. (2023), exposure to high temperature led to a significant increase in global DNA methylation levels in heat-sensitive rice genotypes, whereas methylation levels declined in heat-tolerant genotypes, no significant change was observed in moderately tolerant lines. Consistently, a greater number of hypomethylation events were detected in the tolerant group, while hypermethylation events were more prevalent in the sensitive group (Li et al. 2023). Polyploidization further modifies genomic composition and gene expression, and phenotypic variation and physiological traits in polyploid plants are closely linked to epigenetic regulation (Scarrow et al. 2021; Liu et al. 2022). In the present study, polyploidization was associated with increased DNA methylation, whereas heat stress induced widespread hypomethylation. The overall methylation patterns of CG, CHG, and CHH contexts changed in the same direction, suggesting that these methylation types may share common regulatory pathways during polyploidization and heat adaptation. Methylation profiling across functional regions revealed clear differences among samples, not only in methylation levels but also in their genomic distribution. High CG methylation was primarily enriched in introns and repetitive elements, CHG methylation was concentrated largely in repetitive regions, and CHH methylation showed elevated levels in promoter regions and repetitive sequences. This distribution pattern suggests that different methylation types may carry distinct regulatory responsibilities in gene expression. For instance, in rice, cytosine methylation can occur in CG, CHG, or CHH sequence contexts, with dynamic changes in CHH methylation being most frequently associated with stress responses and growth-related processes (Abid et al. 2017; Bartels et al. 2018). In a study by He et al. 2020 cytosine methylation and demethylation events across the rice genome were found to occur predominantly in the CG context, whereas CHH-context methylation was closely associated with nighttime high-temperature stress. In particular, coordinated methylation or demethylation of consecutive cytosines within promoter or downstream regions of genes involved in ABA-related oxidative responses and ROS scavenging was shown to enhance heat tolerance in rice. In peanut, CHH methylation modulates genes involved in fatty acid biosynthesis and lipid metabolism during seed development (Yu et al. 2025b), illustrating that each methylation context can display a unique response pattern depending on genotype, tissue type, or environmental conditions. The distinct distribution and responsiveness observed in this study therefore likely reflect functional specialization among methylation types in regulating gene activity under both polyploidization and heat stress.

### Methylation dynamics coordinate hormone biosynthesis and signaling under heat stress

Plant hormones serve as central regulators of defence responses (Shafqat et al. 2024). Among them, JA, an endogenously synthesized lipid-derived hormone, has been shown to play a critical role in rice thermotolerance (Yadav et al. 2025). JA biosynthesis begins in the chloroplast, where alpha-linolenic acid released from chloroplast membranes undergoes a series of enzymatic reactions that ultimately generate JA (Li et al. 2021). Tryptophan functions as a precursor for several important secondary metabolites, including IAA. Previous work demonstrated that IAA enhances heat resistance in cucumber by strengthening photosystem performance and activating DNA repair pathways (Yu et al. 2025a). ETH production is also induced by a wide range of abiotic stresses, and its signaling pathway is involved in plant defence against multiple environmental challenges. In *Arabidopsis*, IAA and ETH act synergistically to maintain root growth under high-temperature conditions (Fei et al. 2019).

In the present study, analysis of hormone biosynthesis and signaling pathways revealed that key genes in the auxin pathway (*Os02g0592000, Os11g0515500*) and a major regulator of ETH signaling (*Os03g0700800*) exhibited strong negative correlations between CG methylation in exonic regions and gene expression. This pattern suggests that heat stress promotes the synthesis of JA, IAA, and ETH in association with methylation changes at the alpha-linolenic acid, tryptophan, and cysteine metabolic pathways, thereby enhancing the plant's adaptive capacity to elevated temperatures.

### Heat-induced hypomethylation enhances ROS-scavenging capacity in tetraploid rice

The dynamic balance of ROS in plant cells is determined by the equilibrium between their production and scavenging (Glorieux et al. 2024). Excessive ROS accumulation leads to oxidative damage of cellular membranes, disrupting their structure, impairing function, and reducing permeability (Quan et al. 2008). In the present study, DAB and NBT staining revealed higher levels of H_2_O_2_ and superoxide anions (O_2_^−^) in the leaves of GFD-2X than in GFD-4X (Figs. 1g and 1h), consistent with the observed physiological indicators of oxidative stress. CAT, SOD, and POD play essential roles in ROS detoxification and in the maintenance of metabolic and signaling homeostasis (Li et al. 2024a). Heatmap analysis of antioxidant enzyme-related genes (Fig. S4) showed that the expression levels of these genes were significantly higher in GFD-4Xthan in GFD-2X. Moreover, several key antioxidant enzyme-encoding genes, including *OsSODA1*, *OsAPX1*, *Osprx62*, and *OsCatB,* were continuously induced under heat stress, indicating that GFD-4X can more effectively activate and sustain the transcriptional regulation of antioxidant defense pathways. In addition, the expression of *Os04g0628200*, a peroxisome-associated gene involved in ROS scavenging, was strongly upregulated after heat treatment, and CG hypomethylation was detected within its intronic region. This pattern suggests that reduced methylation under high-temperature conditions may enhance peroxisomal function to mitigate ROS accumulation, thereby supporting cellular homeostasis during heat stress.

### HSP gene activation is facilitated by intronic hypomethylation under heat stress

High temperatures exert profound effects on plant growth, metabolism, and productivity, largely by causing protein misfolding and denaturation (Zhou et al. 2023). HSPs play a central role in protecting cells from such damage. They recognize exposed hydrophobic amino acids on nonnative proteins, promote the folding or refolding of denatured polypeptides, assist in the assembly of protein complexes, and ensure the proper trafficking and sorting of proteins. HSPs also participate in the regulation of the cell cycle and signaling pathways under elevated temperatures (Guanglong et al. 2019). In the present study, genes such as *Os07g0192300, Os01g0355500*, and *Os05g0181000* exhibited CG hypomethylation within intronic regions, and their expression levels were negatively correlated with methylation. These observations suggest that heat stress enhances the expression of HSP-related genes by reducing methylation, thereby alleviating heat-induced cellular stress. Taken together, the results indicate that rice likely engages a highly coordinated regulatory network in response to heat stress. Dynamic adjustments in DNA methylation appear to modulate hormone biosynthesis and signaling pathways, HSP gene expression, and ROS-scavenging systems. Through targeted methylation changes in key functional regions, downstream genes involved in JA, auxin, and ETH biosynthesis, as well as HSP synthesis and ROS detoxification, can be activated to confer enhanced thermotolerance. Notably, these methylation changes were more pronounced in GFD-4X than in GFD-2X, consistent with its stronger physiological and molecular responses to heat stress.

### Advances in transcriptional activity and epigenetic potential underlying heat-stress responses in polyploid rice

The molecular mechanisms by which rice responds to heat stress involve multiple coordinated processes, including cell membrane lipid remodeling, maintenance of physiological homeostasis, antioxidant enzyme–mediated regulation of ROS, and the repair of damaged proteins by HSPs (Yin et al. 2024). In the heat-tolerant rice cultivar Nagina 22, heat stress has been shown to specifically activate JAZ genes through DNA demethylation, leading to elevated JAZ expression and consequent repression of JA signaling, thereby enhancing heat tolerance (Du et al. 2025). The heat shock transcription factor *OsHsfc1a* acts as a positive regulator of thermotolerance in rice seedlings. Heat stress strongly induces *OsHsfc1a* expression, which in turn represses *OsMFT1* transcription, *OsMFT1* negatively affects the maintenance of chloroplast structural integrity, promotes chloroplast damage, and reduces seedling survival under high-temperature conditions (Lu et al. 2026).

In our study, heat stress triggered hypomethylation of key stress-responsive genes in the tetraploid line GFD-4X, thereby regulating the accumulation of phytohormones, antioxidant metabolites, and HSPs. These ploidy-dependent differences in transcriptional activity suggest that genome doubling confers additional regulatory capacity, enhancing the epigenetic and transcriptional plasticity available for stress adaptation.

Previous work by Song et al. (2025a) demonstrated that moderate cold priming of parental rice plants induces specific alterations in DNA methylation patterns. Compared with non-primed controls, these plants exhibited enhanced cold tolerance, which was stably inherited by subsequent generations. This finding indicates that rice can transmit acquired cold tolerance to its progeny *via* DNA methylation–mediated epigenetic inheritance, enabling transgenerational transmission of acquired stress resilience (Song et al. 2025a). Although transgenerational epigenetic inheritance has been reported in rice under other stress contexts, the present study focuses on short-term stress and recovery responses. In the present study, we observed that following a high-temperature recovery phase, the expression levels and metabolite contents of several hormone- and antioxidant enzyme–related genes remained elevated in GFD-4X (Fig. 3). This suggests that physiological and biochemical traits associated with heat adaptation are partially retained in the tetraploid line, potentially enabling a more rapid and effective response to recurrent heat stress during later growth stages.

Overall, these findings provide insights into the molecular mechanisms underlying plant adaptation to high temperatures and offer important clues to the epigenetic basis of heat tolerance in polyploid rice. The identified pathways and gene targets hold potential value for crop improvement strategies aimed at enhancing resilience to heat and other abiotic stresses. By integrating physiological, transcriptomic, and methylomic data without reliance on single-gene perturbation, this study provides a system-level framework rather than causal dissection of individual epigenetic regulators.

## Materials and methods

### Plant materials and heat treatment

The japonica diploid rice line GFD-2X and its corresponding autotetraploid GFD-4X were used as experimental materials. The latter originated as a naturally occurring mutant of GFD-2X during cultivation and exhibits stable agronomic traits, it has been self-pollinated for seven generations (Leng et al. 2023). All plants were grown hydroponically in 0.5× Kimura B nutrient solution under controlled environmental conditions in a growth chamber, maintained at 26 °C/20 °C (day/night) with a 16 h/8 h (light/dark) photoperiod.

Seedlings at the three-leaf stage with uniform growth were selected for a temperature-gradient assay to determine the optimal treatment temperature. Plants were exposed to 36 °C, 38 °C, 40 °C, 42 °C, and 44 °C for seven days. Both GFD-2X and GFD-4X seedlings exhibited leaf rolling and wilting at elevated temperatures. Based on the severity of morphological responses—severe necrosis at 42 °C and above, and only mild effects at 38 to 40 °C was selected as the optimal temperature for subsequent heat-stress experiments (Fig. S5).

Heat treatment was applied for 7 d, followed by a 7 d recovery period under normal growth conditions. At both time points, GFD-2X and GFD-4X seedlings were harvested for phenotypic measurements, including plant height, root length, fresh weight, dry weight, and water-loss rate. Sampling was performed for heat-treated, recovery, and corresponding control groups, each with three biological replicates. For each replicate, stem-and-leaf tissues were collected from randomly selected groups of 20 individuals after 7 d of heat or recovery treatment and immediately frozen in liquid nitrogen. Samples were processed for RNA-seq and MethylC-seq analyses following established protocols (Song et al. 2017).

### Histochemical staining and quantitative analysis ROS accumulation in rice leaves

Histochemical staining was performed to assess the accumulation of O_2_^−^ and H_2_O_2_ using NBT and DAB staining, respectively (Li et al. 2024b). Leaf samples from GFD-2X and GFD-4X seedlings under stress and corresponding control conditions were incubated in DAB solution (1 mg mL^−1^ dissolved in 10 mmol L^−1^ MES buffer, pH 6.5). Samples were vacuum-infiltrated for 15 min and then incubated in darkness for 12 h. After staining, 95% ethanol was added, and the samples were immersed in a boiling water bath for 10 min. Upon cooling, the tissues were further decolorized in fresh 95% ethanol until the solution became clear.

For NBT staining, a freshly prepared NBT solution (0.5 mg mL^−1^, pH 7.8) stored in darkness was used. Leaf samples were vacuum-infiltrated in the NBT solution for 15 min and subsequently incubated for 30 min. Decolorization was carried out in 95% ethanol for 24 to 48 h until the solution became colorless. The stained leaves were photographed to document the distribution and intensity of ROS accumulation. Quantitative analysis of DAB and NBT staining was conducted using ImageJ software (version 1.54) (Schindelin et al. 2012).

### Determination of antioxidant activity and hormone contents

The contents of antioxidant-related physiological indicators—including H_2_O_2_ (microplate assay), O_2_^−^ (visible spectrophotometry), SOD (WST-8 method), POD (enzyme-linked assay), CAT (UV absorption method), MDA (enzyme-linked assay), soluble proteins (BCA method), soluble sugars (enzyme-linked assay), proline (enzyme-linked assay), and ROS (fluorescence staining)—were measured using commercial assay kits according to the manufacturers' protocols. Transcription rate was measured using a Yaxin-1101S portable photosynthesis system (Beijing Yaxin, Beijing, China).

Phytohormone contents, including auxins (IAA, IBA; HPLC), ETH (HPLC), ABA (high-performance liquid chromatography, HPLC), SA (spectrophotometry), JA (HPLC), tZ (HPLC), and GA (immunoassay), were determined using the corresponding detection kits, following the instructions provided by the manufacturers.

### RNA isolation and RNA-seq analysis

Total RNA was extracted using TRIzol reagent (Invitrogen, Carlsbad, CA, USA), and RNA concentration was measured with a NanoDrop spectrophotometer (Thermo Fisher Scientific, Waltham, MA, USA). For cDNA library construction, mRNA was purified using Dynabeads Oligo(dT) (Thermo Fisher Scientific), randomly fragmented, and reverse-transcribed into complementary DNA (cDNA). After synthesis of double-stranded cDNA, end repair, A-tailing, adaptor ligation, and PCR amplification were performed following the standard Illumina library preparation protocol (Sanfilippo et al. 2017). Sequencing was carried out on the Illumina NovaSeq 6000 platform (Illumina, San Diego, CA, USA). Raw reads were filtered to remove adaptor-containing sequences, reads with ambiguous bases (N), and low-quality reads. Clean reads were aligned to the rice reference genome (NIP-T2T.fa, http://www.ricesuperpir.com) using HISAT2 (version 2.2.1) for rapid and accurate mapping (Kawahara et al. 2013). Transcript abundance was quantified as FPKM using the Subread package (Liao et al. 2013).

Differential expression analysis was performed using the DESeq2 package (version 1.22.1) in R (version 4.4.2). DEGs were identified using the criteria: count > 30, |log_2_ fold change| > 1, and adjusted *P* < 0.05. Identified DEGs were subjected to GO enrichment analysis using the DAVID database (https://davidbioinformatics.nih.gov/).

## Weighted gene co-expression network construction

WGCNA was performed using the WGCNA package (v1.6.9) in R (v4.4.2) (Langfelder and Horvath 2008). DEGs with COUNT values greater than 30 in at least one sample were selected for network construction. The parameters used in the WGCNA workflow were as follows: variance in gene expression > 0, proportion of missing expression data < 0.1, soft-thresholding power = 12, maximum block size = 1,000, deep split = 4, minimum module size = 30, and merge cut height = 0.25 (DiLeo et al. 2011; Zhao et al. 2024). Pearson correlation coefficients were calculated between module eigengenes (MEs) and physiological traits of GFD-2X and GFD-4X to identify modules with significant trait associations. Modules exhibiting the strongest correlations were designated as hub modules. Within these modules, genes with a gene significance (|GS|) > 0.20 and a module membership (|MM|) > 0.91 were classified as hub genes (Azam et al. 2023).

### WGBS-seq analysis

Genomic DNA was extracted using the cetyltrimethylammonium bromide (CTAB) method (Allen et al. 2006). A total of 100 ng genomic DNA per sample was used for library preparation. Unmethylated cytosines were converted to uracil using the EZ DNA Methylation-Gold™ Kit (Zymo Research, Irvine, CA, USA), followed by adaptor ligation, fragment size selection, and PCR amplification to complete library construction. Libraries passing quality control were sequenced on the Illumina platform (Illumina, San Diego, CA, USA) to generate 150 bp paired-end reads. Raw sequencing data were assessed for quality using FastQC (version 0.11.8). After removal of low-quality reads and adaptor sequences, clean data were obtained. Bisulfite-treated reads were aligned to the reference genome using Bismark (version 0.24.0; Krueger et al. 2011) with the parameters “-X 700 -dovetail” (Krueger and Andrews 2011). DMRs were identified using the DSS package (version 2.12.0; Feng et al. 2014; Wu et al. 2015; Park et al. 2016).

Parameters for DMR calling were set as follows: smoothing.span = 200, delta = 0, minlen = 50, minCG = 3, p.threshold = 1 × 10^−5^, dis.merge = 100, pct.sig = 0.5, and abs(diff.Methy) > 0.4 for CG context, > 0.3 for CHG context, and > 0.1 for CHH context (Feng et al. 2014; Wu et al. 2015; Park and Wu, 2016).

DMRs were classified into gene-body regions (from transcription start site, TSS, to transcription end site, TES) and promoter regions (0 to 2 kb upstream of TSS) based on their genomic locations.

### Quantitative reverse transcriptase PCR analysis

Total RNA (1 μg) was used for cDNA synthesis with a reverse transcription kit (Vazyme, Nanjing, Jiangsu, China) following the manufacturer's instructions. Gene expression levels were quantified using RT-qPCR, with actin used as the internal reference gene (Fig. S6). Relative expression values were calculated using the comparative Ct method, and 3 biological replicates were included for each sample (Caldana et al. 2007).

Amplification was performed under the following cycling conditions: initial denaturation at 95 °C for 10 min, followed by 50 cycles of denaturation at 95 °C for 25 s, annealing at 58 °C for 25 s, and extension at 72 °C for 20 s. Primer sequences used for RT-qPCR analysis are listed in Table S7.

### Statistical analysis

Statistical analyses were performed using SPSS software (version 22.0; IBM Corp., Armonk, NY, USA). Significant differences were determined by one-way analysis of variance (ANOVA) followed by Tukey's test (*P* < 0.05). Values with *P* < 0.05 and *P* < 0.01 were considered statistically significant and highly significant, respectively. Data visualization was conducted using the online platform Microbioinformatics (https://www.bioinformatics.com.cn/) and R software (version 4.4.2).

### Accession numbers

Sequence data from this article can be found in the GenBank data libraries under accession numbers: PRJNA1424358, PRJNA1424546, and PRJNA1425664. All study data are included in the article and/or supporting information.

## Supplementary Material

kiag135_Supplementary_Data

## Data Availability

Raw data for the transcriptome and methylome are available in the NCBI Sequence Read Archive (SRA) under accession numbers PRJNA1424358, PRJNA1424546, and PRJNA1425664.
