# Supplementary material for: Transcriptomic and DNA methylation insights into polyploidy-enhanced heat tolerance in rice (Oryza sativa L.)
Source: Plant Physiol. 2026 Mar 17;200(4):kiag135. doi: 10.1093/plphys/kiag135 (PMC13081708; doi:10.1093/plphys/kiag135)
Supplement: kiag135_Supplementary_Data [file kiag135_supplementary_data.zip › PLPHYS-2025-2310R2_Supplemental_Data_0503.pdf]

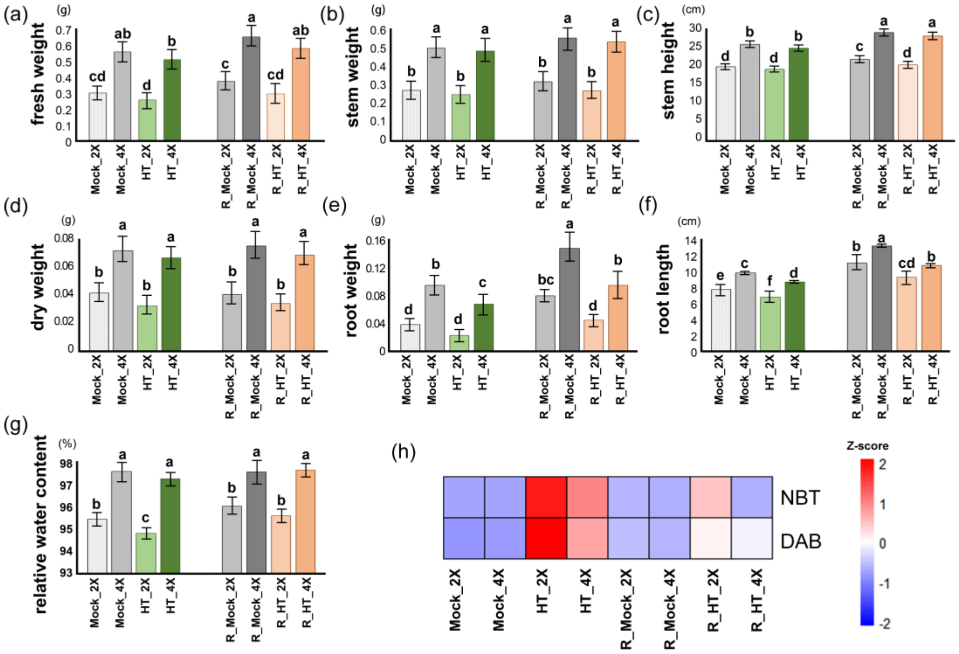

Supplementary Figure S1. Morphological characteristics of diploid (GFD-2X) and tetraploid (GFD-4X) rice seedlings under heat stress. (a) fresh weight, (b) stem weight, (c) stem height, (d) dry weight, (e) root weight, (f) root length, (g) relative water content. The detections were performed with three biological replicates, each biological replicate consisted of 3 technical replicates, with 10 plant strains per replicate. The error bar type is standard deviation. The statistical test method is one-way analysis of variance (ANOVA). (h) Heatmap of NBT and DAB staining intensity data analysis. Different lowercase letters (a–e) indicate statistically significant differences ($P < 0.05$). Mock-2X: Control diploid during stress. Mock-4X: Control tetraploid during stress. HT-2X: High-temperature stressed diploid. HT-4X: High-temperature stressed tetraploid. R_Mock-2X: Control diploid during recovery. R_Mock-4X: Control tetraploid during recovery. R_HT-2X: High-temperature stressed diploid during recovery. R_HT-4X: High-temperature stressed tetraploid during recovery.

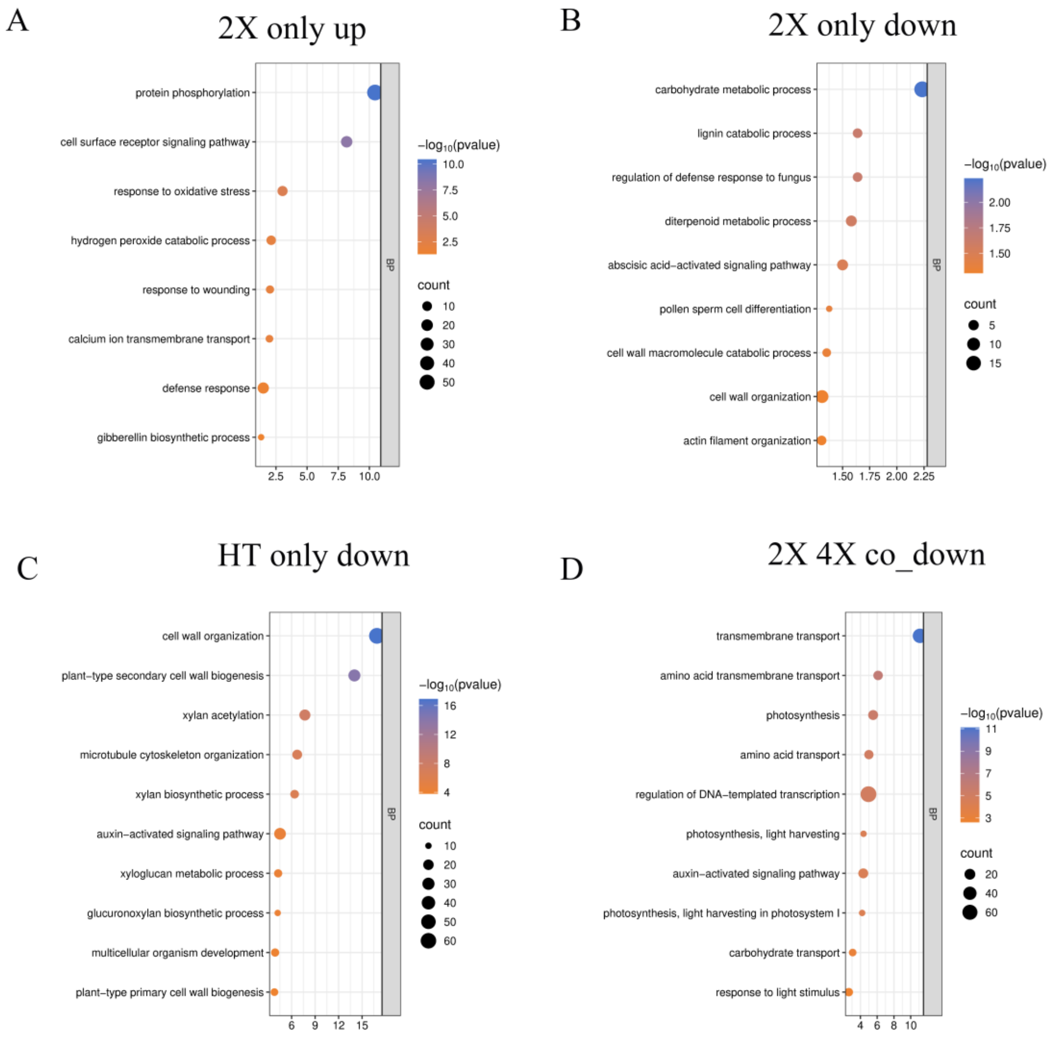

Supplementary Figure S2. Gene Ontology (GO) enrichment analysis results of differentially expressed genes (DEGs). (a) GO enrichment analysis of DEGs specifically upregulated in diploid rice. (b) GO enrichment analysis of DEGs specifically downregulated in diploid rice. (c) GO enrichment analysis of DEGs specifically downregulated under high-temperature treatment. (d) GO enrichment analysis of DEGs co-downregulated in diploid and tetraploid rice. 2X: diploid. 4X: tetraploid.

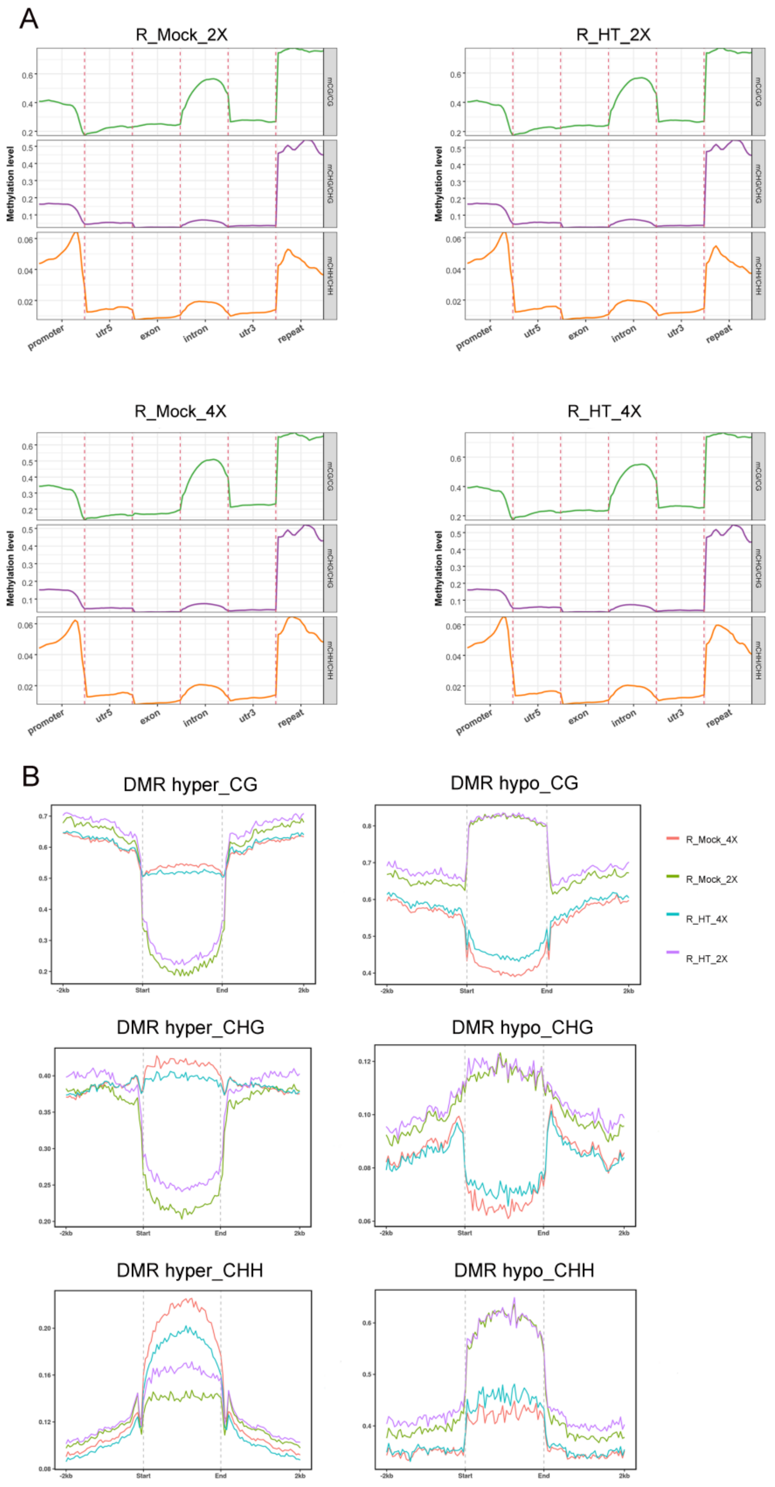

Supplementary Figure S3. Methylation expression patterns of rice under high temperature stress in different ploidy states. (a) Distribution of methylation levels in functional regions. (b) Variations in three methylation types (high, intermediate, low) between diploid and tetraploid rice. The x-axis represents different regions of the gene, and the y-axis represents methylation levels. R_Mock-2X: Control diploid

during recovery. R_Mock-4X: Control tetraploid during recovery. R_HT-2X: High-temperature stressed diploid during recovery. R_HT-4X: High-temperature stressed tetraploid during recovery.

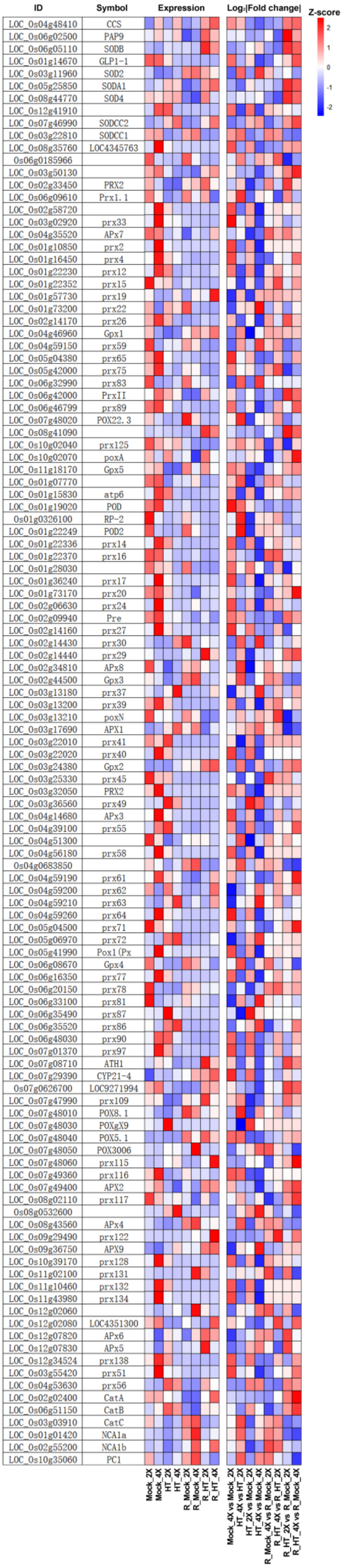
ID
Symbol
Expression
Log2|Fold change|
Z-score
2
1
0
-1
-2
LOC_Os04g48410 CCS
LOC_Os06g02500 PAP9
LOC_Os06g05110 SODB
LOC_Os01g14670 GLP1-1
LOC_Os03g11960 SOD2
LOC_Os05g25850 SODA1
LOC_Os08g44770 SOD4
LOC_Os12g41910
LOC_Os07g46990 SODCC2
LOC_Os03g22810 SODCC1
LOC_Os08g35760 LOC4345763
Os06g0185966
LOC_Os03g50130
LOC_Os02g33450 PRX2
LOC_Os06g09610 Prx1.1
LOC_Os02g58720
LOC_Os03g02920 prx33
LOC_Os04g35520 APx7
LOC_Os01g10850 prx2
LOC_Os01g16450 prx4
LOC_Os01g22230 prx12
LOC_Os01g22352 prx15
LOC_Os01g57730 prx19
LOC_Os01g73200 prx22
LOC_Os02g14170 prx26
LOC_Os04g46960 Gpx1
LOC_Os04g59150 prx59
LOC_Os05g04380 prx65
LOC_Os05g42000 prx75
LOC_Os06g32990 prx83
LOC_Os06g42000 PrxII
LOC_Os06g46799 prx89
LOC_Os07g48020 POX22.3
LOC_Os08g41090
LOC_Os10g02040 prx125
LOC_Os10g02070 poxA
LOC_Os11g18170 Gpx5
LOC_Os01g07770
LOC_Os01g15830 atp6
LOC_Os01g19020 POD
Os01g0326100 RP-2
LOC_Os01g22249 POD2
LOC_Os01g22336 prx14
LOC_Os01g22370 prx16
LOC_Os01g28030
LOC_Os01g36240 prx17
LOC_Os01g73170 prx20
LOC_Os02g06630 prx24
LOC_Os02g09940 Pre
LOC_Os02g14160 prx27
LOC_Os02g14430 prx30
LOC_Os02g14440 prx29
LOC_Os02g34810 APx8
LOC_Os02g44500 Gpx3
LOC_Os03g13180 prx37
LOC_Os03g13200 prx39
LOC_Os03g13210 poxN
LOC_Os03g17690 APX1
LOC_Os03g22010 prx41
LOC_Os03g22020 prx40
LOC_Os03g24380 Gpx2
LOC_Os03g25330 prx45
LOC_Os03g32050 PRX2
LOC_Os03g36560 prx49
LOC_Os04g14680 APx3
LOC_Os04g39100 prx55
LOC_Os04g51300
LOC_Os04g56180 prx58
Os04g0683850
LOC_Os04g59190 prx61
LOC_Os04g59200 prx62
LOC_Os04g59210 prx63
LOC_Os04g59260 prx64
LOC_Os05g04500 prx71
LOC_Os05g06970 prx72
LOC_Os05g41990 Pox1(Px
LOC_Os06g08670 Gpx4
LOC_Os06g16350 prx77
LOC_Os06g20150 prx78
LOC_Os06g33100 prx81
LOC_Os06g35490 prx87
LOC_Os06g35520 prx86
LOC_Os06g48030 prx90
LOC_Os07g01370 prx97
LOC_Os07g08710 ATH1
LOC_Os07g29390 CYP21-4
Os07g0626700 LOC9271994
LOC_Os07g47990 prx109
LOC_Os07g48010 POX8.1
LOC_Os07g48030 POXgX9
LOC_Os07g48040 POX5.1
LOC_Os07g48050 POX3006
LOC_Os07g48060 prx115
LOC_Os07g49360 prx116
LOC_Os07g49400 APX2
LOC_Os08g02110 prx117
Os08g0532600
LOC_Os08g43560 APx4
LOC_Os09g29490 prx122
LOC_Os09g36750 APX9
LOC_Os10g39170 prx128
LOC_Os11g02100 prx131
LOC_Os11g10460 prx132
LOC_Os11g43980 prx134
LOC_Os12g02060
LOC_Os12g02080 LOC4351300
LOC_Os12g07820 APx6
LOC_Os12g07830 APx5
LOC_Os12g34524 prx138
LOC_Os03g55420 prx51
LOC_Os04g53630 prx56
LOC_Os02g02400 CatA
LOC_Os06g51150 CatB
LOC_Os03g03910 CatC
LOC_Os01g01420 NCA1a
LOC_Os02g55200 NCA1b
LOC_Os10g35060 PC1
Mock_2X
Mock_4X
HT_2X
HT_4X
R_Mock_2X
R_Mock_4X
R_HT_2X
R_HT_4X
Mock_4X vs Mock_2X
HT_4X vs HT_2X
HT_2X vs Mock_2X
HT_4X vs Mock_4X
R_Mock_4X vs R_Mock_2X
R_HT_4X vs R_HT_2X
R_HT_2X vs R_Mock_2X
R_HT_4X vs R_Mock_4X

Supplementary Figure S4. Heatmap showing the expression of antioxidant enzyme gene. Twenty plants were pooled together as one sample in each replicate. All samples were tested in three independent experiments with three replicates each. Mock-2X: Control diploid during stress. Mock-4X: Control tetraploid during stress. HT-2X: High-temperature stressed diploid. HT-4X: High-temperature stressed tetraploid. R_Mock-2X: Control diploid during recovery. R_Mock-4X: Control tetraploid during recovery. R_HT-2X: High-temperature stressed diploid during recovery. R_HT-4X: High-temperature stressed tetraploid during recovery.

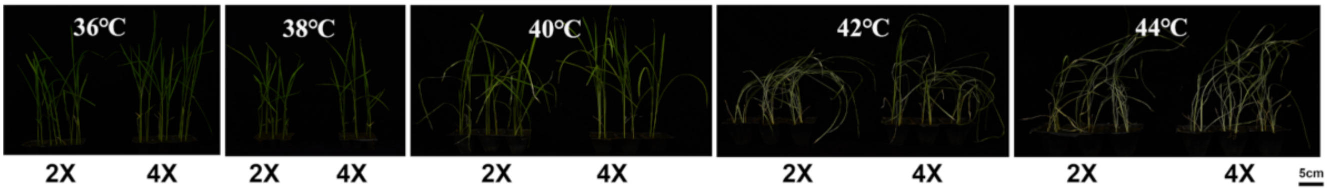

Supplementary Figure S5. Morphological changes of diploid and tetraploid rice after 7 days of cultivation at 36, 38, 40, 42 and 44 ℃. Bar = 5 cm. 2X: diploid. 4X: tetraploid.

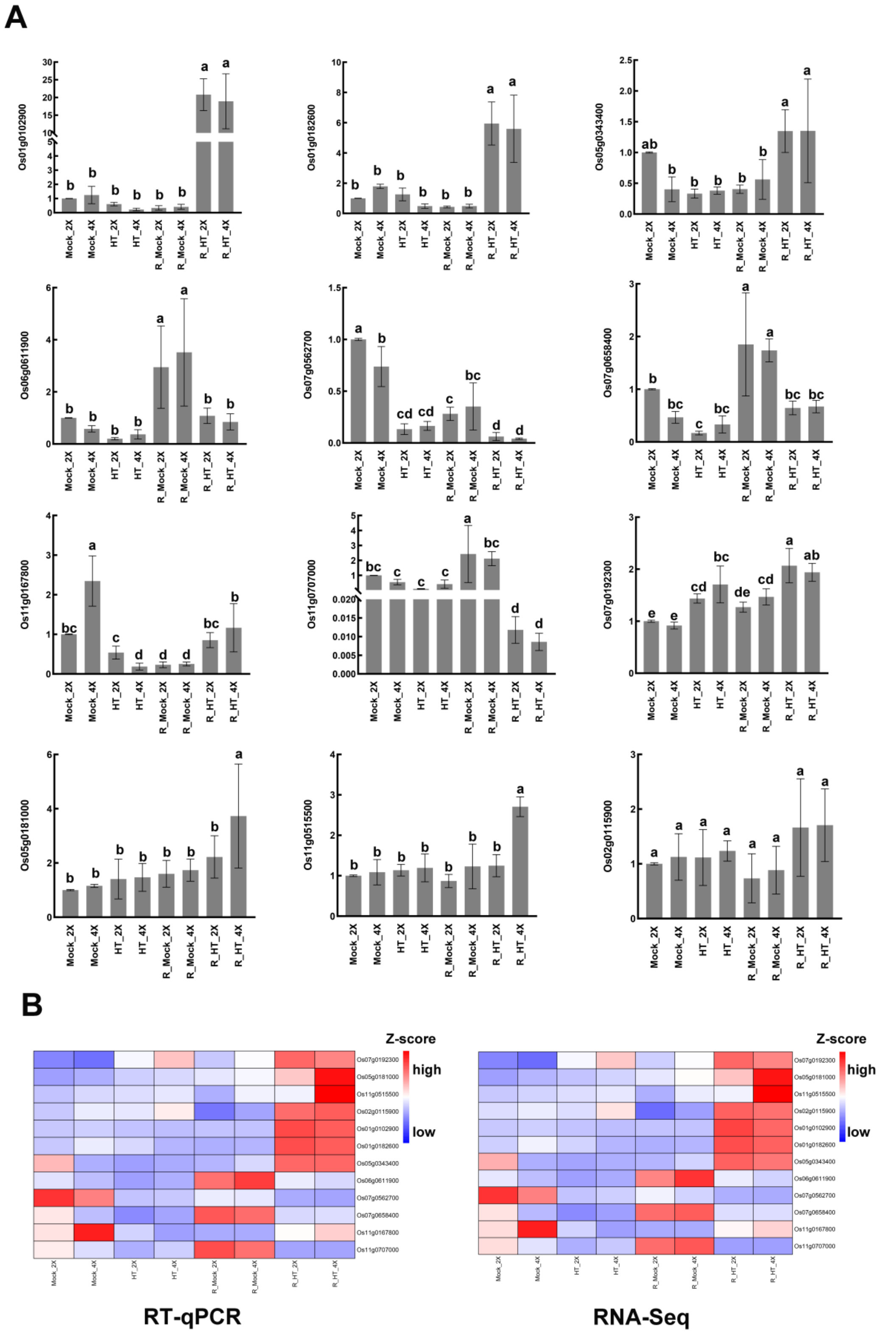

Supplementary Figure S6. Verification of gene expression by comparing transcriptome and RT-qPCR. (a) Reverse transcription-quantitative PCR (RT-qPCR) results for 12 genes. (b) Comparison of RNA-seq and RT-qPCR data. The detections were performed with three biological replicates, each biological replicate consisted

of 3 technical replicates, with 20 plant strains mixed per replicate. The error bar type is standard deviation. The statistical test method is one-way analysis of variance (ANOVA). Different lowercase letters (a–d) indicate statistically significant differences ($P < 0.05$). Mock-2X: Control diploid during stress. Mock-4X: Control tetraploid during stress. HT-2X: High-temperature stressed diploid. HT-4X: High-temperature stressed tetraploid. R_Mock-2X: Control diploid during recovery. R_Mock-4X: Control tetraploid during recovery. R_HT-2X: High-temperature stressed diploid during recovery. R_HT-4X: High-temperature stressed tetraploid during recovery.
